# A convenient model of serum-induced reactivity of human astrocytes to investigate astrocyte-derived extracellular vesicles

**DOI:** 10.3389/fncel.2024.1414142

**Published:** 2024-06-10

**Authors:** Katherine E. White, Hannah L. Bailey, Barry S. Shaw, Philippine C. Geiszler, Raquel Mesquita-Ribeiro, Daniel Scott, Robert Layfield, Sébastien Serres

**Affiliations:** ^1^School of Life Sciences, University of Nottingham, Nottingham, United Kingdom; ^2^College of Science, University of Derby, Derby, United Kingdom; ^3^The David Greenfield Human Physiology Unit, University of Nottingham, Nottingham, United Kingdom

**Keywords:** astrocytes, serum, reactivity, extracellular vesicles, proteomics

## Abstract

Extracellular vesicles (EVs) are secreted by all cells in the CNS, including neurons and astrocytes. EVs are lipid membrane enclosed particles loaded with various bioactive cargoes reflecting the dynamic activities of cells of origin. In contrast to neurons, the specific role of EVs released by astrocytes is less well understood, partly due to the difficulty in maintaining primary astrocyte cultures in a quiescent state. The aim of this study was to establish a human serum-free astrocyte culture system that maintains primary astrocytes in a quiescent state to study the morphology, function, and protein cargoes of astrocyte-derived EVs. Serum-free medium with G5 supplement and serum-supplemented medium with 2% FBS were compared for the culture of commercially available human primary fetal astrocytes. Serum-free astrocytes displayed morphologies similar to *in vivo* astrocytes, and surprisingly, higher levels of astrocyte markers compared to astrocytes chronically cultured in FBS. In contrast, astrocyte and inflammatory markers in serum-free astrocytes were upregulated 24 h after either acute 2% FBS or cytokine exposure, confirming their capacity to become reactive. Importantly, this suggests that distinct signaling pathways are involved in acute and chronic astrocyte reactivity. Despite having a similar morphology, chronically serum-cultured astrocyte-derived EVs (ADEVs) were smaller in size compared to serum-free ADEVs and could reactivate serum-free astrocytes. Proteomic analysis identified distinct protein datasets for both types of ADEVs with enrichment of complement and coagulation cascades for chronically serum-cultured astrocyte-derived EVs, offering insights into their roles in the CNS. Collectively, these results suggest that human primary astrocytes cultured in serum-free medium bear similarities with *in vivo* quiescent astrocytes and the addition of serum induces multiple morphological and transcriptional changes that are specific to human reactive astrocytes and their ADEVs. Thus, more emphasis should be made on using multiple structural, molecular, and functional parameters when evaluating ADEVs as biomarkers of astrocyte health.

## Introduction

Astrocytes are the most abundant glial cells in the central nervous system (CNS) and responsible for a wide range of functions, including neurotrophic support and synaptic maintenance through neurotransmitter recycling and gliotransmission, all being essential to maintain brain homeostasis (Andersen and Schousboe, 2023). Another key role of astrocytes is their ability to change into reactive phenotypes to aid microglia to mediate neuroinflammation in response to injury or pathology (Liddelow and Barres, 2017; Escartin et al., 2021). Whilst acute astrocyte reactivity is important for safeguarding healthy brain function, chronic reactivity is often observed in neurodegeneration and thought to exacerbate disease progression and neuronal death (Brandebura et al., 2023). In addition to loss of neuroprotection, neurotoxic secretions are released from reactive astrocytes that actively cause neuronal death (Guttenplan et al., 2020, 2021; Arredondo et al., 2022).

One of the main challenges with neurodegenerative diseases is the length of time it takes to reach a diagnosis, which means that patients cannot access essential support or begin possible treatments until later in the disease progression. Given that astrocyte reactivity typically emerges in the early stages of disease, astrocyte secretions hold promise as a biomarker source for early diagnosis and disease stratification (Escartin et al., 2019).

Extracellular vesicles (EVs) are lipid membrane bound particles containing proteins, nucleic acids, and metabolites, released from almost all cells and which are thought to represent the state-of-the cell at the time of release (Couch et al., 2021). Therefore, it is hypothesized that astrocyte-derived EVs (ADEVs) would display differences between quiescent and reactive phenotypes that could potentially be used as external biomarkers of astrocyte reactivity in CNS. A clinical benefit of ADEVs is their ability to cross the blood-brain barrier (BBB) so they could be harvested from blood samples, allowing non-invasive diagnostic tests in the future (Willis et al., 2017; Rouillard et al., 2021). EVs have already been shown to carry pathological proteins such as mutant SOD1 and α-synuclein suggesting ADEVs may also have an active role in the transmission of neurodegenerative diseases (Lian et al., 2015; Silverman et al., 2019; Stuendl et al., 2021; Zhao et al., 2021).

In recent years, the field has begun to understand the importance of characterizing ADEVs with studies undertaking proteomic and transcriptomic analyses in astrocyte models (Dickens et al., 2017; Chaudhuri et al., 2018; You et al., 2020). However, a limitation with research into ADEVs, so far, is the lack of physiologically relevant models of both quiescent and reactive astrocytes, which heavily depend on the use of rodent tissues to culture primary astrocytes. While being useful to understand astrocytic functions *in vitro*, rodent models do not necessarily translate into human biology with key phenotypic differences between rodent and human astrocytes (Oberheim et al., 2006, 2009). Moreover, primary cultures typically use fetal bovine serum (FBS)-containing medium, which supports the growth and proliferation of astrocytes (McCarthy and De Vellis, 1980), but also changes their morphology and characteristics (Prah et al., 2019). In addition to growth factors, proteins, and vitamins, FBS contains significant levels of lipopolysaccharides and endogenous EVs that have been shown to alter astrocyte biology and phenotype (Aswad et al., 2016). Besides, bovine EVs present in FBS can contaminate EV preparations and potentially make the analysis of bioactive cargoes found in human ADEVs meaningless (Aswad et al., 2016; Lehrich et al., 2018). Finally, astrocytes would not be exposed to components of serum in an intact BBB, and once mature, they should not proliferate under healthy conditions.

Overall, this raises questions as to whether primary astrocytes maintained in serum cultures should be used as a reliable *in vitro* model of quiescent astrocytes. Instead, it is thought that astrocytes obtained using FBS culture system are in the reactive state (Du et al., 2010), and to improve astrocyte culture purity and the expression of astrocyte-specific proteins, efforts are being made in developing serum-free primary astrocyte culture systems (Codeluppi et al., 2011; Foo et al., 2011). A new method combining antibody-based immunopanning methods and serum-free medium containing supplement has been shown to mimic astrocyte phenotypes *in vivo* (Foo et al., 2011; Zhang et al., 2016). However, this method is time-consuming, very expensive and not without caveats when isolating purified astrocytes from the dishes (Prah et al., 2019).

Serum should be absent from quiescent astrocyte models, yet reliable and reproducible serum-free culture systems for human primary astrocytes are currently unavailable. With a simple serum-free method to culture human primary astrocytes, it would be therefore possible to mimic the *in vivo* brain microenvironment, as well as offering translational value when evaluating ADEVs as clinical biomarkers of astrocyte reactivity in CNS pathologies.

Here, we cultured commercially available human primary astrocytes in an optimized serum-free medium using G5 supplement, to mimic quiescent conditions, and compared their morphological and transcriptional changes to treatments with either 2% FBS or cytokines using immunocytochemistry, RT q-PCR, western blotting, and RNA sequencing. ADEVs from both serum and serum-free media were isolated to study their morphology and function, and proteomic analysis was used for characterizing protein cargoes in ADEVs.

## Methods

### Serum-cultured astrocytes

Human primary astrocytes (SC1800s) were purchased from ScienCell (Cat# SC-1800; distributed by Caltag Medsystems, UK) and stored in liquid nitrogen (−196°C) prior to use. For serum-cultured conditions, cells were cultured according to the supplier's instructions. In brief, astrocytes were cultured in astrocyte medium (ScienCell, distributed by Caltag Medsystems, UK) supplemented with 2% FBS, 1% astrocyte growth serum (AGS) and 1% penicillin/streptomycin solution (P/S) and maintained at 37°C in a humidified atmosphere of 5% CO_2_. Cells were seeded at a density of 4 × 10^3^ cells/cm^2^ and passaged every 3–4 days until a maximum of 7 passages.

### Serum-free astrocyte culture

To culture serum-free astrocytes, thawed cells from the supplier were initially cultured with serum for a single passage to encourage proliferation before cryopreserving in liquid nitrogen (-196°C) in a solution of 90% FBS (Sigma, UK) and 10% dimethyl sulfoxide (DMSO; Sigma, UK). Upon subsequent revival, cells were spun down and thawed in serum-free medium [advanced DMEM (Gibco, UK)] supplemented with 1% G5 supplement (Fisher scientific, UK), 1% L-glutamine (Sigma, UK) and 1% P/S (Sigma, UK) with a medium change occurring after 24 h to remove any residual serum from the cryopreservation. Due to a lack of proliferation, cells were seeded at a high density (1.6 × 10^4^ cells/cm^2^) and sub-culturing was not required. Instead, a medium change was performed every 3–4 days.

### Short-term (acute) treatment of serum-free astrocyte culture with 2% FBS or cytokines

Serum-free astrocytes were treated with either 2% FBS (Caltag, UK) or an inflammatory cytokine cocktail for 24 h. The cytokine cocktail was made up of recombinant human IL-1α at 100 μg/mL, TNFα at 50 μg/mL (both Peprotech, UK) and C1q at 400 ng/mL (Merck, UK), which had previously been shown to induce an inflammatory phenotype in astrocytes (Liddelow et al., 2017).

### Extracellular vesicle isolation from astrocytes

#### Medium collection for EV isolation

To harvest sufficient EVs for downstream approaches, at least four collections of medium were completed from serum-free astrocytes (over 2 weeks) and combined for processing. Due to the long collection time, conditioned medium was preserved at −80°C until needed. For serum-cultured cells, FBS needed to be removed from the medium before ADEVs were collected to avoid contamination of bovine EVs. To this end, cells were seeded as usual in astrocyte medium containing 2% FBS. After 24 h, the cells were carefully washed with PBS before the astrocytes were cultured in astrocyte medium without FBS with the reactive phenotype being maintained. The conditioned medium was then collected after 72 h and stored at −80°C until needed. A minimum of 4x T75 flasks of medium were required to harvest enough EVs for downstream processing.

#### Ultrafiltration and size exclusion chromatography for EV isolation

The conditioned medium (~48 mL) was concentrated to 500 μL through centrifugation using 10 kDa MWCO protein concentrators (ThermoFisher scientific, UK) at 3,600 × g. The concentrated 500 μL was then added to a washed qEV original 70 nm column (IZON, France). Three fractions were collected according to the manufacturer's instructions. Between samples, the column was washed with both PBS and 0.5 M NaOH solution equal to 2 column volumes. Fractions were frozen at −80°C for longer term storage when required. If a more concentrated EV solution was required for downstream applications, EV fractions were combined and centrifuged using 3 kDa MWCO protein concentrators (Merck, UK) until the desired volume was required (>10-fold concentration).

### EV physical characterization

To visualize EVs, EV-enriched fractions were fixed in 3% glutaraldehyde and loaded onto copper carbon-coated, 200-mesh electron microscopy grids (EM resolutions, UK). Grids were then imaged on the Tecnai Spirit Bio-twin T12 transmission electron microscope (TEM). Nanoparticle tracking analysis (NTA) was completed on the Zetaview^®^ (Analytik, UK) to measure the size and concentration of the EVs within the EV-enriched fractions.

### EV treatment of astrocytes

The three EV fractions obtained from SEC were combined before measuring EV concentration using NTA. EVs were diluted to 2.5 – 5.0 × 10^11^ particles/mL. Serum-free astrocytes were then treated with either 10 μL (2 × 10^9^ particles) or 100 μL (2 × 10^10^ particles) of purified reactive ADEVs for 24 h.

### Immunocytochemistry

Astrocytes were plated onto 13 mm coverslips at a density of 1 × 10^5^ cells/coverslip for serum-cultured or 2 × 10^5^ cell/coverslip for serum-free astrocytes in a 24-well plate and incubated at 37°C and 5% CO_2_ for 48 h. Cells were fixed in 4% paraformaldehyde, permeabilised for 10 min using 0.1% (v/v) Triton X-100 in PBS and then blocked using 1% (w/v) bovine serum albumin (BSA) for 1 h at room temperature. Cells were then labeled overnight with either anti-GFAP (rabbit, 1:500, Dako, UK), anti-EAAT2 (mouse, 1:500, Santa Cruz biotechnology, USA) or anti-S100β (rabbit, neat, Dako, UK) in 1% BSA at 4°C. For immunofluorescence detection, either anti-rabbit Alexa Fluor 488 (goat, 1:500, Invitrogen, UK), anti-mouse Alexa Fluor 488 (goat, 1:500, Invitrogen, UK) or anti-rabbit Alexa Fluor 568 (goat, 1:500, Invitrogen, UK) was applied for 1 h at room temperature, shielded from light. After washing with PBS three times, the coverslips were then mounted onto glass slides using VectorShield mounting medium containing 4,6-diamidino-2-phenylindole (DAPI; VectorShield, UK) and stored at 4°C until imaged. Images were taken using an inverted fluorescent microscope equipped with a Camera Axiocam 305 monodigital (Axio Vert A1, Zeiss microscopy, UK). Image analysis was then completed using FIJI software (Image J, NIH, USA).

### Morphological analysis of astrocytes

Live cell imaging was performed using an inverted microscope (AXIO Vert A.1, Zeiss microscopy, UK) with a 10x lens (Zeiss microscopy, UK). Images were processed to assess cell morphology and growth using FIJI software. Briefly, morphological analysis consisted of manually drawing around individual cells to measure both area and perimeter of cells. The area/perimeter ratio was then calculated to account for the diversity in astrocyte morphology seen in culture and to reduce the effect that long processes would have on cell area (i.e., measurements focused on change in soma size). Additionally, the size of astrocyte nuclei was also assessed using DAPI staining for cell senescence or maturation (Heckenbach et al., 2022).

#### Real-time qPCR

Total RNA was extracted from the astrocytes using TRIzol^®^ (ThermoFisher Scientific, UK) and quantified using the 2000c UV/IV Spectrophotometer (Nanodrop; ThermoFisher Scientific, UK) before synthesis of complementary DNA (cDNA) using Superscript III reverse transcriptase (ThermoFisher Scientific, UK). Quantitative real-time PCR (RT-qPCR) was performed using PowerUp™ SYBR™ Green master-mix (ThermoFisher Scientific, UK) and either the StepOne Real time PCR system (Applied Biosystems, UK) or QuantStudio 5 (ThermoFisher Scientific, UK). RT-qPCR amplification was completed using the following protocol: 95°C for 10 min followed by 40 cycles of 95°C for 15 s, 60°C for 20 s and 72°C for 35 s. To verify the specificity of the amplification, a melt curve was generated after the amplification. Data were normalized to the global mean of all primers and the fold change calculated using 2^−ΔΔ*Ct*^ (Soto et al., 2016). Primer sequences are included in Supplementary Table 1.

### RNA-sequencing

Total RNA was extracted as above using TRIzol^®^ from both serum-free and serum-cultured astrocytes and quantified using the 4200 TapeStation system (Agilent, UK). RNA-sequencing was then performed by Novogene Advances Genomics services (Novogene, UK). Briefly, FPKM (Fragments Per Kilobase per Million mapped fragments) in RNA seq-data were obtained. Unreliably quantified data, defined as more than 25% of unquantified data per group, were discarded. The remaining data were log2 transformed, and a threshold of −15 was applied before normalization to the median ensuring comparability between groups. Then, zFPKM values were calculated from the normalized data using a threshold of zFPKM > −3 to select expressed genes only (Hart et al., 2013). The 16830 most expressed genes were used to run the multivariate statistical analysis.

Multivariate statistical analysis was performed on mean-centered and autoscaled zFPKM data using SIMCA (v.18, Sartorius Stedim Data Analytics, Sweden). Principal component analysis (PCA) was used to highlight distinct patterns in the data and partial least squares discriminant analysis (PLS-DA) to reveal which genes were responsible for group differences. To determine the potential predictive value of the models, a Q^2^ (cum) value was calculated for each model, where a Q^2^ ≥ 0.5 being regarded as good (Broadhurst et al., 2018) and ≥ 0.7 as robust (Salek et al., 2010).

### Immunoblotting

To harvest astrocytes for western blotting, the medium was removed, and cells were washed with PBS. Cells were scraped and harvested in fresh PBS. Cells were pelleted by centrifugation at 1,500 × g for 5 min at 4°C. PBS was removed, and cells were lysed for 15 min in RIPA lysis buffer (5 M sodium chloride, 0.5 M EDTA (pH 8.0), 1 M Tris (pH 8.0), 1% (v/v) NP40, 10% (w/v) sodium deoxycholate, 10% (w/v) SDS) with protease and phosphatase inhibitors at a dilution of 1:1000, followed by sonication for 10 s. Cell debris was pelleted and removed by centrifugation at 16,100 × g for 20 min 4°C. The supernatants were collected to calculate protein concentration using a Pierce BCA assay kit (ThermoFisher Scientific, UK).

Samples of 1–10 μg protein were heated for 5 min with 5 μL gel application buffer (0.15 M Tris, 8 M urea, 2.5% (w/v) SDS, 20% (v/v) glycerol, 10% (v/v) 2-mercaptoethanol, 3% (w/v) DTT), then separated using a 5%-20% polyacrylamide gradient SDS-PAGE gel. Proteins were transferred overnight to a nitrocellulose membrane (AmershamTM Protran^®^ western blotting membrane, 0.45 μm pore, Fisher Scientific, UK) in Western Transfer Buffer (25 mM Tris, 192 mM glycine and 20% (v/v) methanol) at 40 mA and 500 V.

The membrane was washed 3x in Tris-buffered saline (TBS; 20mM Tris, 150mM NaCl, pH 7.5) with 0.1% (v/v) Tween (TBS-T) before blocking in 5% milk-TBS-T (5% (w/v) Marvel milk powder in TBS-T) for 1 h at room temperature. The membrane was then incubated with primary antibody diluted in 5% milk-TBS-T overnight at 4°C. Mouse anti-GFAP (1:2,000, Abcam, UK), mouse anti-EAAT2 (1:500, Santa Cruz biotechnology, USA), mouse anti-GAPDH (1:5,000, Proteintech, UK) and mouse anti-HSP70 (1:1,000, GeneTex, USA) were used as primary antibodies. Primary antibodies were washed with 3x TBS-T, then a secondary peroxidase rabbit anti-mouse antibody (1:3,000, Dako, UK) in blocking buffer was incubated with the membrane for 1 h. After 2x TBS-T washes and 1x TBS wash, a 1:1 mix of Western Lightning Plus ECL reagents (enhanced chemiluminescence, Perkin Elmer, UK) was incubated with the membrane for ~1 min. Finally, the membrane was developed on film and fixed. Western-blot densitometry of the film was then performed using FIJI software (Image J, NIH, USA).

### Proteomic analysis of astrocytes and ADEVs

#### Sample preparation

For proteomic analysis, protein extracts were prepared as for immunoblotting. For EV preparations, the EV fraction with the most particles (i.e., EV fraction 2) was heated with 4x Laemmli sample buffer (Bio-Rad, UK) at 95°C for 5 min. All samples were electrophoresed on a 5%−20% gradient SDS-PAGE gel until the sample had passed through the stacking gel and had entered at least 0.5 cm of the resolving gel, maintaining the proteome in a single gel band. The entire gel was stained with Coomassie dye (Bio-Rad, UK) to highlight the region of the gel to be cut where the protein content for each sample migrated. Samples of serum-free and serum-cultured human astrocytes, and corresponding EV fractions purified from conditioned media, were analyzed independently using LC-MS/MS (Cambridge Center for Proteomics; Cambridge, UK; *N* = 3).

#### Proteomic analysis

Generation and analysis of proteomic LC-MS/MS data was used to catalog purified proteins within polyacrylamide gel slices, using tryptic digestion. Coomassie-stained protein bands from SDS PAGE were excised, reduced, alkylated, digested with trypsin, and submitted to MS/MS on an LTQ Orbitrap spectrometer with nanoflow liquid chromatography (LC). Peptides were identified in data-dependent mode, using Mascot (2.7.0) to search the human The UniProt Consortium (2021) and Mellacheruvu et al. (2013) databases (93734 proteins, 2 max missed cleavage sites, precursor ion mass tolerance 20 PPM, fragment ion mass tolerance 0.100 Da) and validated with Scaffold 5 (Proteome Software, Inc., USA). Carbamidomethyl (+57 on C) was considered a fixed modification, while deamidation (+1 on N/Q), oxidation (+16 on M), phosphorylation (+80 on S/T/Y), and acetylation (+42 on K/N-terminal M) were considered as variable modifications. For individual protein identifications, our criteria were a minimum of two unique peptides within a run, with peptide threshold set at 95% and protein threshold 99%, giving a mean FDR (false-discovery rate) of ~17% across runs. Data were filtered for protein detection, i.e. “presence” and “absence,” across biological repeats. Recognizing the common issue of complexity and variation (or stochasticity) in sampling between LC-MS/MS runs, for inclusion in subsequent comparisons, protein detection in at least 2/3 biological repeats was applied.

### Statistical analysis

Graphical data were completed using GraphPad prism (GraphPad Software, Inc., USA). Data were presented as mean ± SEM. Unpaired *t*-test was used to compare data between serum-free and serum conditions. One-way ANOVA followed by Dunnett's multiple comparison test was used to compare the effect of treatments on serum-free astrocytes. One-way ANOVA followed by Tukey's multiple comparison test was used to compare the ranging effect of FBS and EVs on serum-free astrocytes. For differential gene expression analysis, the normalized DESeq method was performed using DESeq2 software, as shown previously (Love et al., 2014). A negative binomial distribution model was used to calculate significance followed by the Benjamini–Hochberg procedure, with a differential gene screening threshold of log2 (FoldChange) ≥ 1 and padj ≤ 0.05.

## Results

### FBS causes a morphological change in human primary astrocytes and promotes cell maturation

Cell adhesion, survival and growth of serum-free astrocyte cultures were initially assessed using published medium formulations (Supplementary Table 2). Of the 5 commonly used serum-free medium formulations, we found that advanced DMEM supplemented with 1% G5 supplement, 1% L-glutamine and 1% P/S was the optimal condition to promote cell adhesion and survival, as well as maintaining a quiescent astrocyte phenotype after 4 weeks in culture (Supplementary Figure 1A).

**Figure 1:**
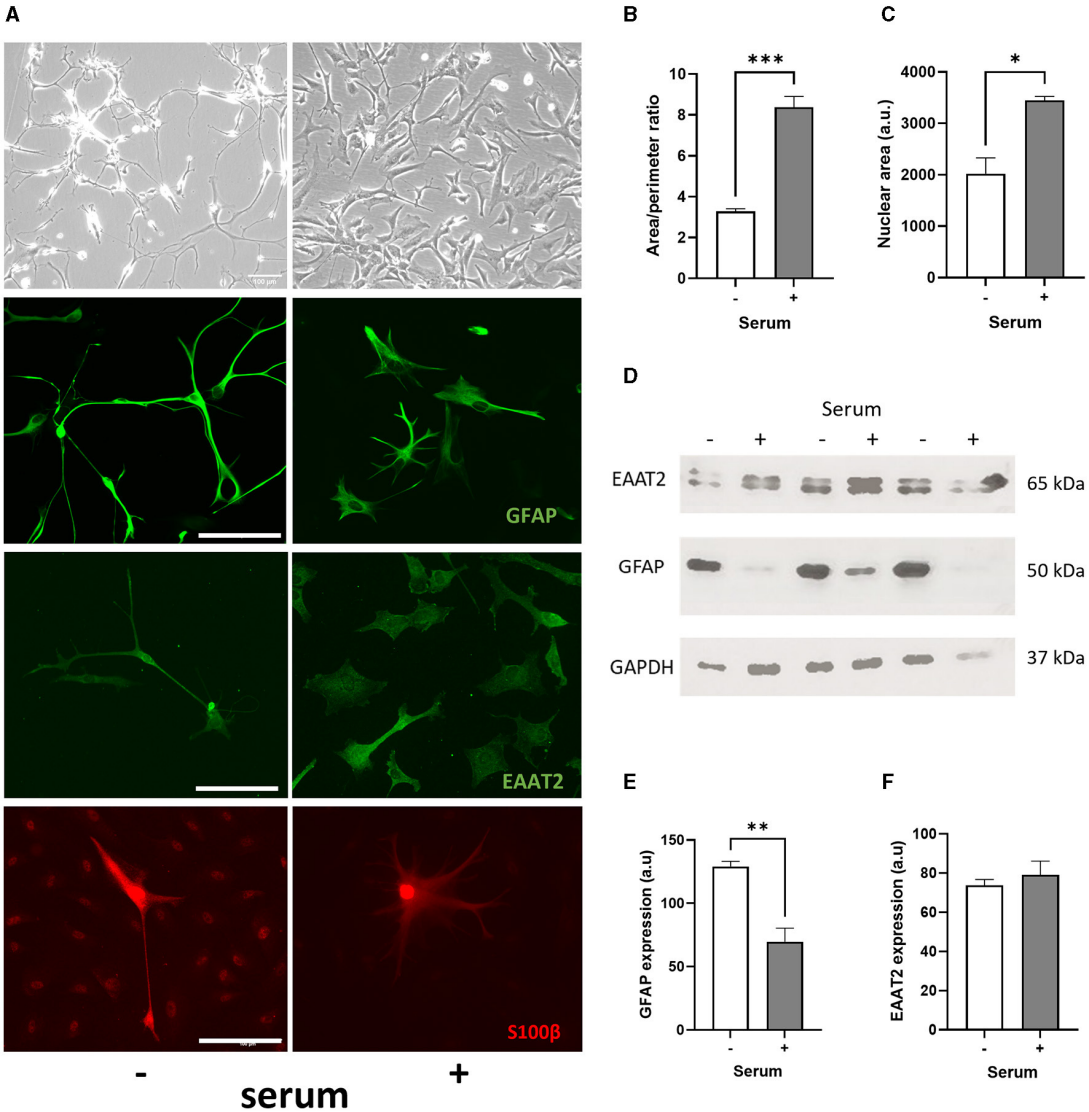
Serum culture causes a morphological change in human primary astrocytes and promotes cell maturation. **(A)** Representative brightfield images (first row), immunofluorescence images of GFAP (second row), EAAT2 (third row) and S100β (fourth row) showing morphological differences between serum-free (–, left column) and serum-cultured astrocytes (+, right column). **(B)** Graphs showing increased area/perimeter ratio (***p < 0.001, *N* = 3, at least 20 cells counted per samples) and **(C)** nuclear size (*p < 0.05, *N* = 3, at least 70 cells counted per samples) for serum-cultured *vs*. serum-free astrocytes. **(D, E)** Immunoblots showing higher GFAP protein levels in serum-free compared to serum-cultured astrocytes (**p < 0.01, *N* = 3), and **(D–F)** similar EAAT2 protein expression in both serum-free and serum-cultured astrocytes. Scale bars represent 100 μm for all images. Data represent mean ± SEM and analyzed using unpaired *t*-test. Measurements were performed after 1 week in culture.

The morphological effect of serum-free culture on human primary astrocytes was then compared to serum condition (2% FBS, 1% AGS, 1% P/S, as recommended by the manufacturer). Morphological changes were evident between both culture conditions, with serum-cultured astrocytes consistently exhibiting hypertrophic cell bodies and smaller processes, whilst serum-free astrocytes had long and thin branching processes (Figure 1A). After 1 week in culture, we found that serum condition increased the area/perimeter ratio of human primary astrocytes 1.5-fold (Figure 1B, *p* < 0.001, *N* = 3), indicating of cellular hypertrophy in reactive astrocytes. In addition to an increase in cell size, a significant increase in nuclear size of astrocytes was observed after 1 week in serum culture compared to serum-free culture, indicating of cell maturation (*ca*. 70%; Figure 1C, *p* < 0.05, *N* = 3). Once astrocytes were exposed to serum, none of these morphological changes could be reversed by removing serum from the medium (Supplementary Figure 1B), as described previously (Prah et al., 2019). However, this contrasted with when the cells were cryopreserved in liquid nitrogen (−196°C) after initial culture in a solution of 90% FBS and 10% DMSO and then revived in serum-free medium with a quiescent phenotype.

### Chronic serum culture induces a reduction in GFAP expression in human primary astrocytes

After 1 week in culture, immunofluorescence staining of GFAP, EAAT2 and S100β in astrocytes revealed the same morphological changes observed in serum-cultured astrocytes (Figure 1A). Surprisingly, GFAP expression per astrocyte was significantly higher in serum-free condition (129 ± 4 vs. 70 ± 11 a.u.; *p* < 0.05, *N* = 3), whilst no significant difference was evident between serum-free and serum-cultured astrocytes for both EAAT2 (128 ± 26 vs. 86 ± 32 a.u.; *N* = 4) and S100β expressions (529 ± 262 vs. 63 ± 26 a.u.; *N* = 4), respectively. Immunoblot analysis further confirmed that serum culture was associated with significant reduction of GFAP protein expression in human primary astrocytes after 1 week (*ca*. 45%; Figures 1D, E, *p* < 0.01, *N* = 3). However, serum culture did not significantly alter EAAT2 protein expression (Figures 1D–F, *N* = 3).

While GFAP is well established as an astrocyte marker, these results confirm that quiescent human primary astrocytes showed similarity with *in vivo* astrocytes, but they do not correlate with increased GFAP expression when astrocytes are chronically cultured in serum, as seen in rodent primary astrocytes (Prah et al., 2019).

### Serum culture of human primary astrocytes downregulates the expression of astrocyte markers and upregulates the expression of inflammatory markers

Quantitative PCR (qPCR) was used to measure the expression of a panel of genes, as used previously to assess reactive phenotype in both rodent and human astrocytes (Foo et al., 2011; Zhang et al., 2016; Prah et al., 2019). Typically, GFAP, S100β, EAAT2 and NDRG2, are expressed in both quiescent and reactive astrocytes. On the other hand, pro-inflammatory cytokines IL-1β and TNFα, and anti-inflammatory marker IL-10 with CD49F integrin receptor are commonly used to assess the inflammatory phenotype of astrocytes (Liddelow et al., 2017; Prah et al., 2019; Barbar et al., 2020; Escartin et al., 2021).

**Figure 2:**
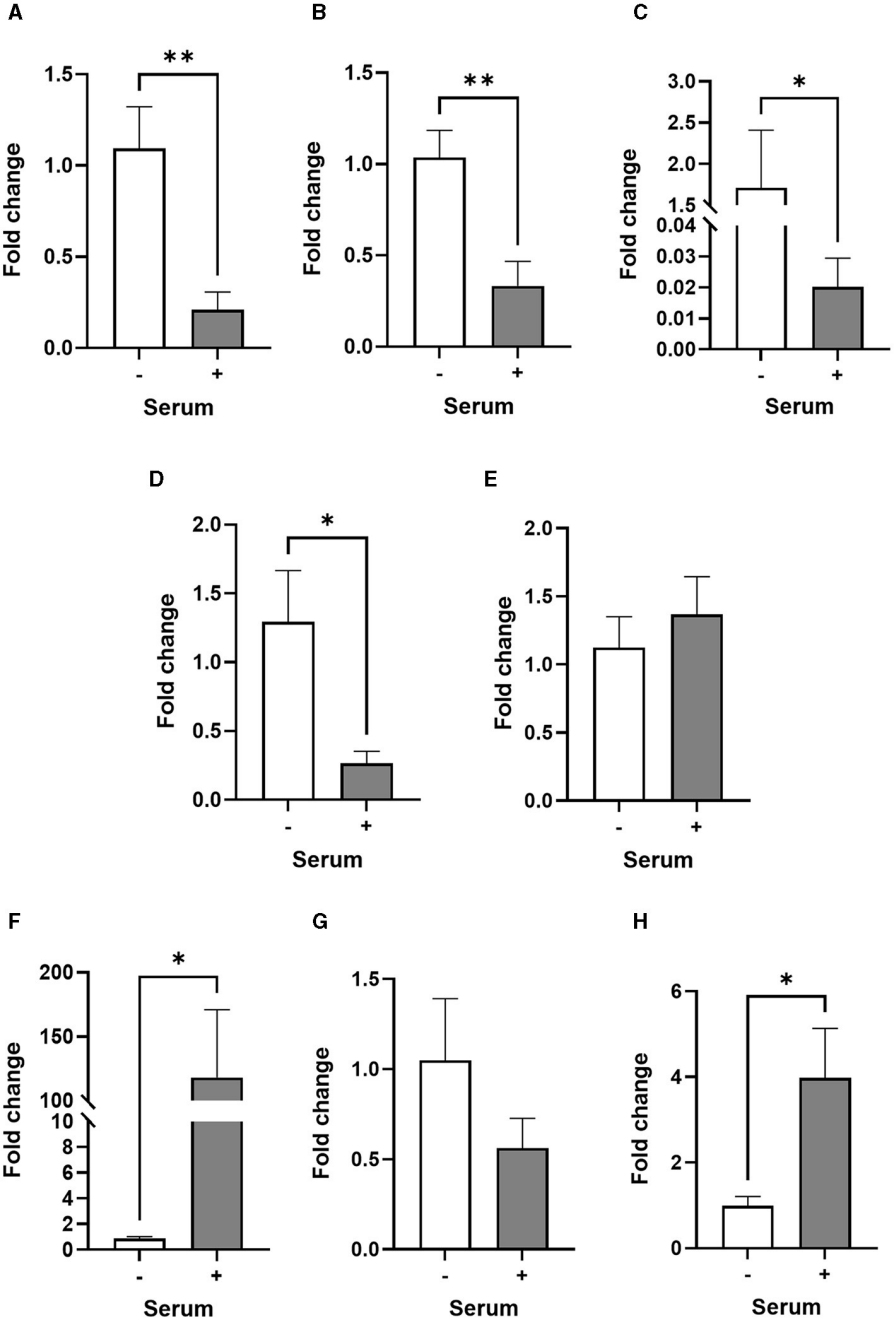
Transcriptional profiles of astrocyte and inflammatory markers are different in serum-free and serum-cultured astrocytes. **(A–D)** Real-time quantitative PCR analysis of GFAP, S100β, EAAT2, and NDRG2 gene expression, respectively, showing downregulation of astrocyte markers in serum-cultured astrocytes compared to serum-free astrocytes (**p < 0.01; *p < 0.05 *vs*. serum-free, *N* = 5). **(E–H)** Real-time quantitative PCR analysis of CD49F, IL-1β, TNF-α and IL-10 gene expression, respectively, showing upregulation of inflammatory markers IL-1β and IL-10 in serum-cultured astrocytes (*p < 0.05 vs. serum free, *N* = 6–8). Data represent mean ± SEM and were analyzed using unpaired *t*-test. Measurements were performed after 1 week in culture.

Surprisingly, the expression of GFAP, S100β, EAAT2 and NDRG2 genes was significantly much lower in serum-cultured astrocytes compared to serum-free cultures after 1 week in culture (*ca*. from 68%−99%; Figures 2A–D). This result suggests that while serum-cultured astrocytes show a reactive cellular morphology, these cells are losing their ability to express astrocyte specific markers upon growth and differentiation. Although the expression of TNFα and CD49F genes were not affected by serum condition, both IL1β and IL-10 genes were consistently upregulated in serum condition (*ca*. from 3 to 134-fold, Figures 2E–H, *p* < 0.05, *N* = 6–8), suggesting that human primary astrocytes cultured in serum display an inflammatory secretory phenotype.

**Figure 3:**
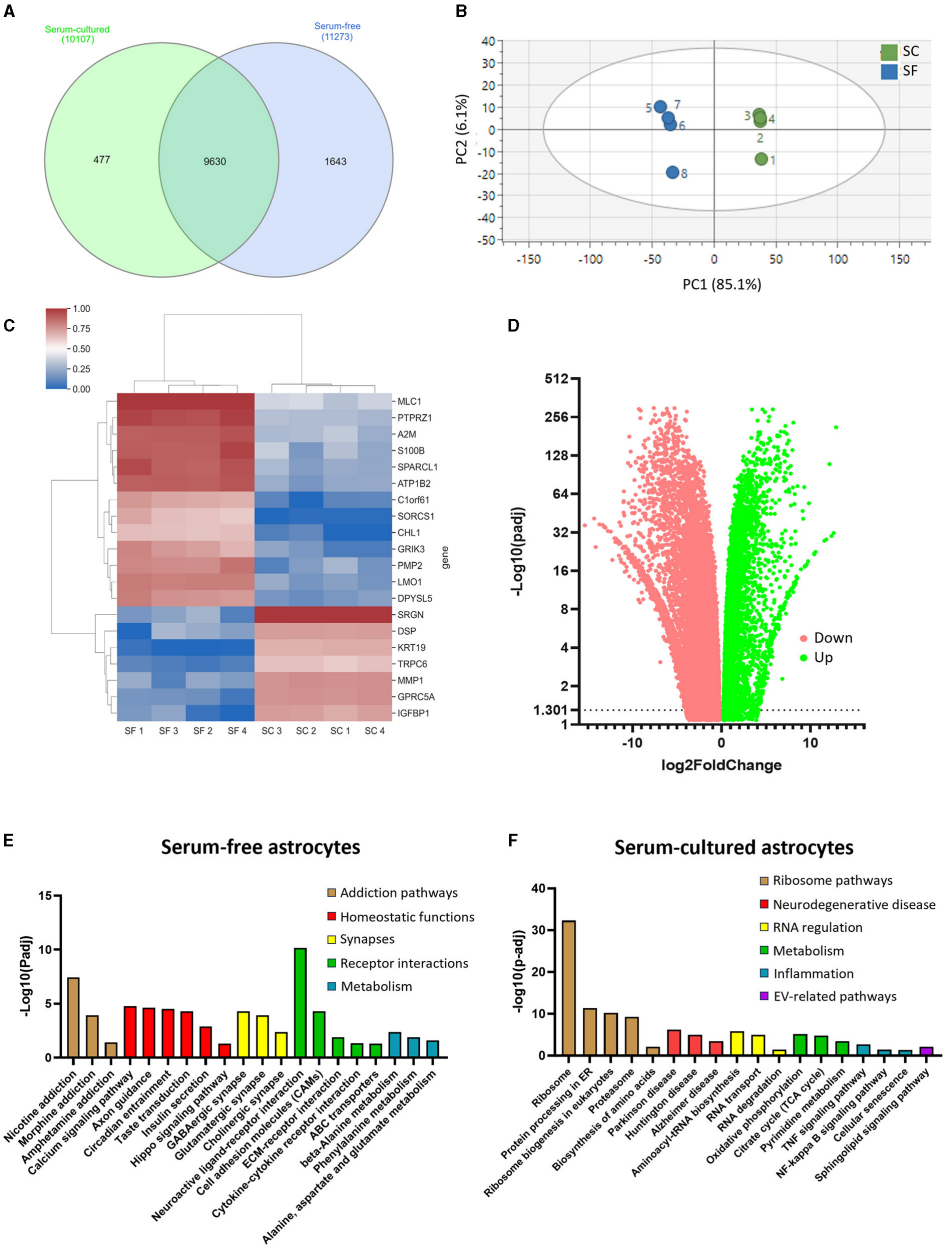
RNA-sequencing analysis reveals distinct transcriptomes for serum-free and serum-cultured astrocytes. **(A)** The majority of protein coding RNA was found in both models, but more unique RNAs were identified in serum-free astrocytes. **(B)** Principal component analysis (PCA) highlights the differential RNA expression between serum-free and serum-cultured astrocytes. **(C)** The expression of the 20 most influential genes identified in the PLS-DA analysis contributing to the separation between serum-free (SF) and serum-cultured astrocyte (SC) transcriptomes. **(D)** Most RNAs identified were differentially expressed between culture conditions with more RNAs downregulated in serum-cultured astrocytes (red) compared to serum-free astrocytes. **(E)** KEGG pathway analysis showing upregulation of supportive pathways such as axon guidance, and synapse associated pathways such as GABAergic synapse and glutamatergic synapse in serum-free astrocytes compared to serum-cultured astrocytes. **(F)** KEGG pathway analysis showing upregulation of CNS disease-associated pathways, as well as increased ribosome-related pathways in serum-cultured astrocytes. Measurements were performed after 1 week in culture.

Having established that serum-free and serum-cultured astrocytes display different morphological and transcriptional profiles, we then used next generation sequencing (NGS) technologies to gain insights into the transcriptome profiles of both cell culture conditions. Most protein-coding gene transcripts were found in both astrocytes with 1,643 genes exclusively expressed in serum-free condition and 477 genes in serum-cultured condition (Figure 3A). PCA was performed to distinguish serum-free and serum-cultured groups and the scores plot showed tight clustering of the four replicates within each group and, most importantly, a clear separation between serum-free and serum-cultured conditions along the first principal component (R^2^X = 0.851 on mean-centered data and R^2^X = 0.560 on auto-scaled data) (Figure 3B). Subsequent PLS-DA confirmed this separation along the first latent variable (R^2^X = 0.851, R^2^Y = 0.995, and Q^2^ = 0.993, mean-centered data). This separation indicates a distinctive gene signature of serum-free and serum-cultured astrocytes. The top 20 genes responsible for this separation were SRGN, KRT19, GPRC5A, IGFBP1, MMP, TRPC6, DSP in serum-cultured astrocytes and CHL1, DPYSL5, GRIK3, A2M, PMP2, LMO, C1orf61, MLC1, S100β, SORCS1, PTPRZ1, SPARCL1, ATP1B2 in serum-free astrocytes (Figure 3C).

Differential gene expression analysis revealed the upregulation of 9367 genes and the downregulation of 6582 genes in serum-free astrocytes compared to serum-cultured astrocytes (Log2 fold change ≥ ± 1; Figure 3D). Evaluation of the list of upregulated genes in serum-free and serum culture models (Supplementary Tables 3, 4) using a KEGG pathway analysis identified 23 pathways that were significantly upregulated in serum-free astrocytes and 46 in serum-cultured astrocytes (Log2 fold change ≥1; Figures 3E, F). Axon guidance and synapse-related pathways were amongst the upregulated pathways in serum-free astrocytes, confirming normal physiological functions of these astrocytes. In contrast, upregulated genes were associated to disease-related pathways such as Parkinson's and Alzheimer's disease, as well as inflammatory pathways (i.e., TNFα and NF-kappa β signaling) in serum-cultured astrocytes, confirming their inflammatory secretory phenotype. Key homeostatic astrocyte functions were also down-regulated in serum-cultured astrocytes such as calcium signaling, axon guidance, glutamatergic and GABAergic synapses, suggesting a reduction in the trophic support of astrocytes for neurons. More genes associated with cellular proliferation were identified in the serum-cultured astrocytes supporting nuclear size increases in serum-cultured condition. Astrocyte-specific genes that were up and downregulated in this analysis (see Supplementary Table 5) were mostly coherent with a previous dataset (Prah et al., 2019).

### Acute treatment with serum or cytokines induces morphological and transcriptional changes in serum-free astrocytes

To further test the ability of serum-free astrocytes to become reactive, astrocytes were acutely treated for 24 h with either 2% FBS or cytokines that are secreted by activated neuroinflammatory microglia (i.e., IL-1α, TNFα and C1q) (Liddelow et al., 2017). The area/perimeter ratio was used to assess morphological changes in both untreated and treated serum-free astrocytes.

**Figure 4:**
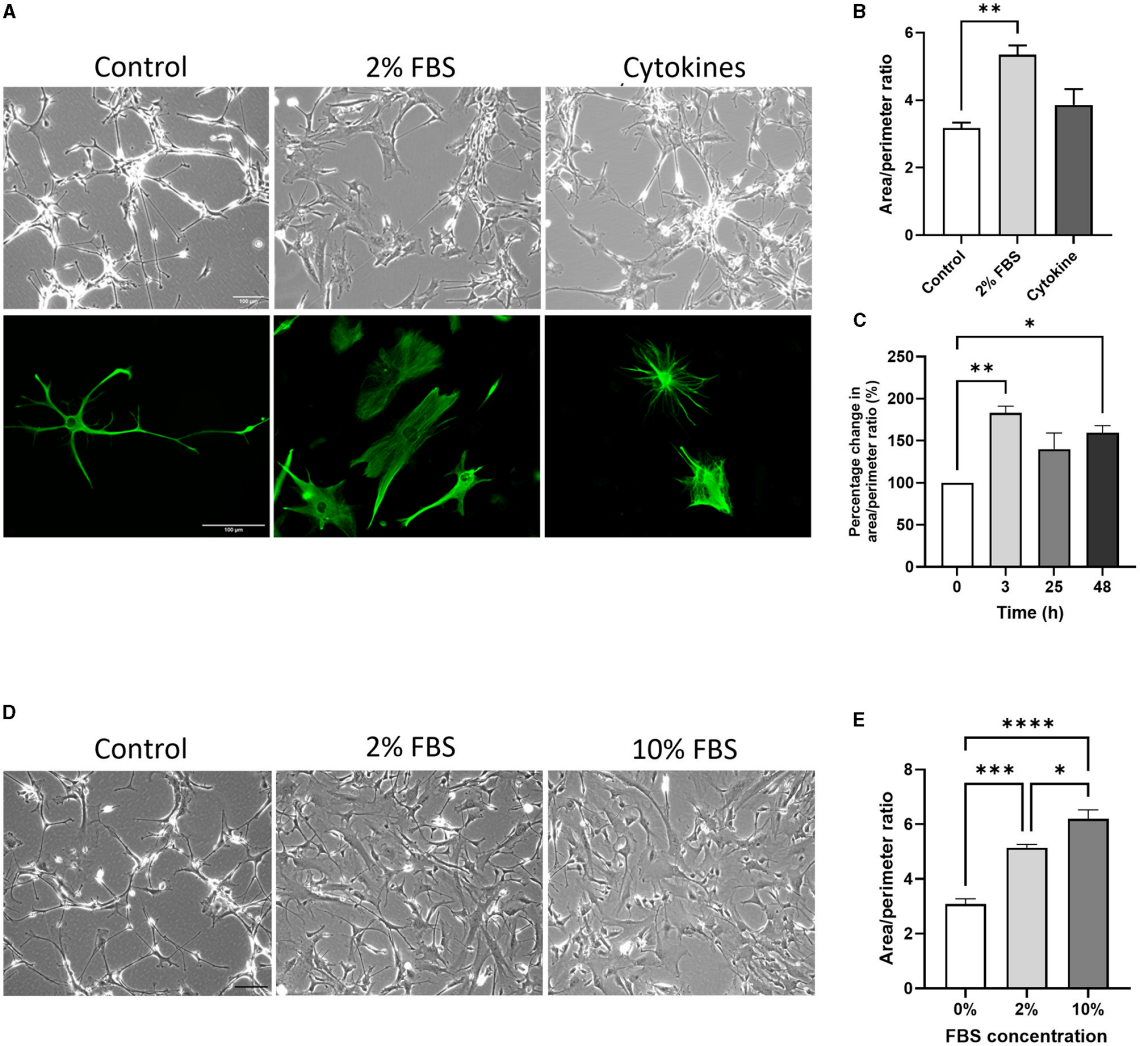
The acute effect of serum and cytokines induces morphological changes in serum-free astrocytes. **(A)** Representative bright field images (top row) and immunofluorescence images of GFAP expression (bottom row) showing morphological differences between untreated (left column), 2% FBS treated (middle column) and cytokine-treated (right column) serum-free astrocytes. **(B)** Morphological analysis showing that 2% FBS significantly increases area/perimeter ratio in serum free astrocytes, whilst cytokine treatment does not (**p < 0.01, *N* = 4–5). **(C)** Graph showing the area/perimeter ratio of serum-free astrocytes in response to 2% FBS over 24 h (*p < 0.05, **p < 0.01, *N* = 3). **(D)** Representative brightfield images of serum-free astrocytes in response to 0, 2 or 10% FBS. **(E)** Morphological analysis showing that FBS dose-dependently increases the area/perimeter ratio of serum-free astrocytes (*p < 0.05, ***p < 0.001, and ****p < 0.0001, *N* = 4). Scale bars represent 100 μm. Data represent mean ± SEM and were analyzed using one-way ANOVA followed by either Tukey's or Dunnett's multiple comparison test. Measurements were performed after 24 h exposure to 2% FBS or cytokines.

A morphological change was evident after 2% FBS treatment for 24 h but not after cytokine treatment on both brightfield and fluorescent images (Figure 4A). Quantitative analysis of astrocyte morphology showed that the area/perimeter ratio of serum-free astrocytes increased significantly in response to 2% FBS treatment (*ca*. 68%; Figure 4B, *p* < 0.01, *N* = 5). Importantly, these morphological changes occurred rapidly (< 3 h) and plateaued after 24 h (Figure 4C, *p* < 0.05- 0.01, *N* = 3). Next, we determined whether the effect of FBS was dose-dependent by assessing morphological changes in serum-free astrocytes following exposure to 2% and 10% FBS in culture medium. There was a significant and gradual increase in area/perimeter ratio from the untreated (0%) condition to 2% and 10% FBS treatments (Figures 4D, E, *p* < 0.01–0.001, *N* = 4).

Similar changes in the expression of astrocyte (GFAP, S100β) and inflammatory specific genes (IL-1β, TNFα) were evident in the acute response to 0, 2 and 10% FBS (Supplementary Figure 2). However, there was no dose-dependent effect of FBS concentration, as seen in the morphological analysis (Supplementary Figure 2).

**Figure 5:**
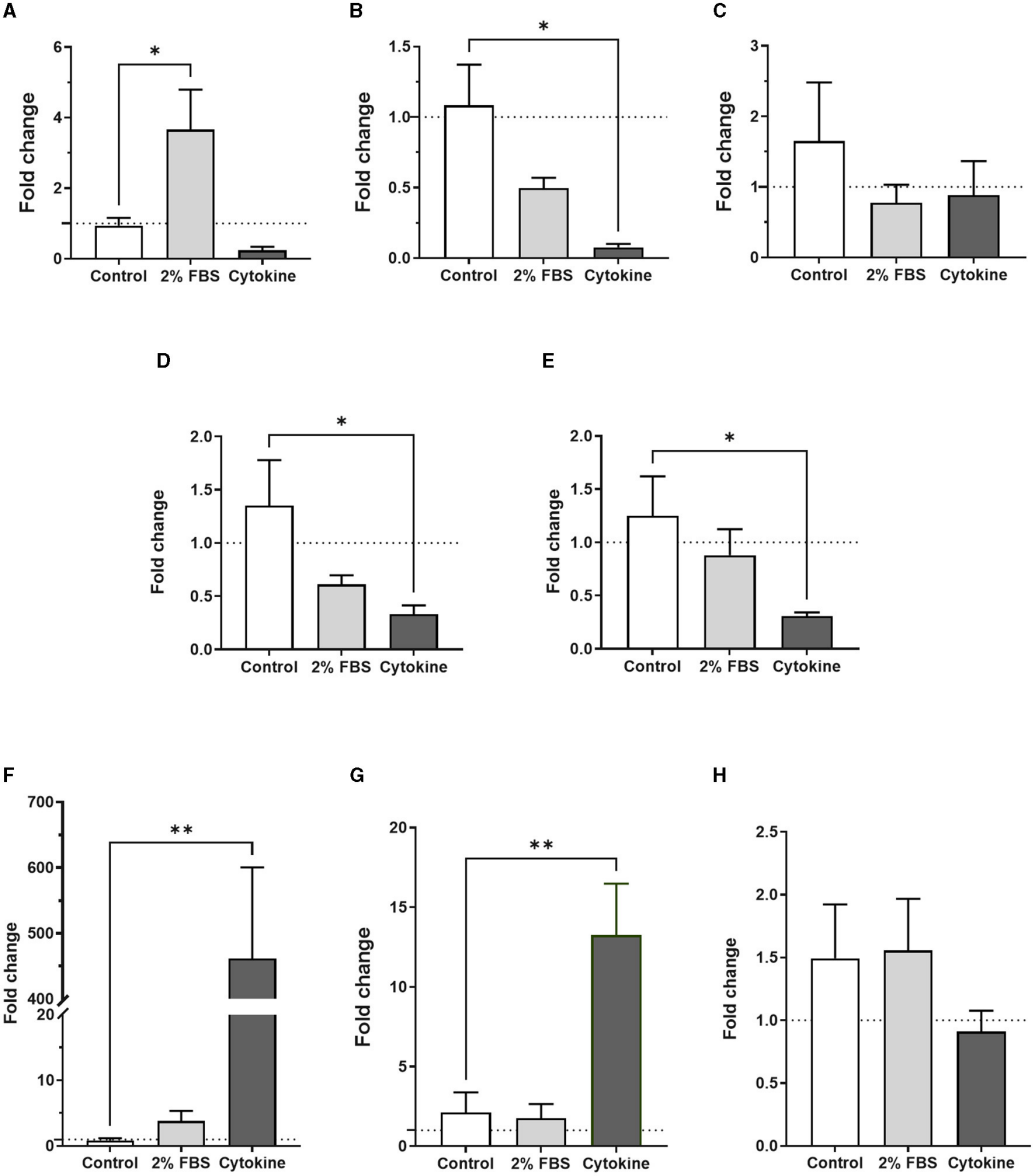
Transcription profiles of astrocyte and inflammatory markers in serum-free astrocytes are different upon acute exposure to either 2% FBS or inflammatory cytokines. **(A)** Real-time quantitative PCR analysis of GFAP expression showing a significant increase after 2% FBS treatment but not after cytokine treatment (*p < 0.05 vs. control, *N* = 6–8). **(B–E)** Real-time quantitative PCR analysis of S100β, EAAT2, NDRG2 and CD49F gene expression, respectively, showing a trend toward downregulation of these markers in cytokine-treated astrocytes. However, only S100β expression was significantly downregulated (*p < 0.05 *vs*. control, *N* = 3–8). No significant difference was identified after acute FBS treatment. **(F–H)** Real-time quantitative PCR analysis of IL-1β, TNF-α and IL-10 gene expression, respectively, showing significant upregulation of inflammatory markers IL-1β and TNF-α in cytokine-treated astrocytes but not in FBS-treated astrocytes (**p < 0.01 vs. control, *N* = 4–8). Data represent mean ± SEM and were analyzed using one-way ANOVA followed by Dunnett's multiple comparison test. Measurements were performed after 24 h exposure to 2% FBS or cytokines.

The same panel of astrocyte and inflammatory specific genes was investigated to assess gene expression in serum-free astrocytes after either cytokine or 2% FBS treatment. Of all astrocyte genes, only GFAP was significantly upregulated in serum-free astrocytes after 2% FBS exposure for 24 h (Figure 5A, *p* < 0.05, *N* = 6–8), in agreement with previous work on rodents (Prah et al., 2019). Although not significant, there was a trend toward downregulation of the same astrocyte genes after 2% FBS exposure (Figures 5B, C, *N* = 3–8). Some astrocyte specific genes were downregulated after acute cytokine exposure (Figure 5, significantly for S100β, NDRG2, and CD49F, *p* < 0.05, *N* = 3–8).

To determine whether 2% FBS or cytokine treatment could induce A1-inflammatory like astrocytes, we measured gene expression of inflammatory markers including IL-1β, TNFα, IL-10 and CD49F in serum-free astrocytes 24 h after 2% FBS treatment. Apart from IL-1β, no inflammatory markers were upregulated in response to 2% FBS (Figure 5, *N* = 8). However, whilst CD49F was downregulated, there was a strong and significant upregulation of pro-inflammatory markers such as IL-1β and TNFα after cytokine treatment (Figures 5F, G, *p* < 0.01, *N* = 8). No significant change was observed for the anti-inflammatory marker IL-10 (Figure 5H, *N* = 8). The protein expression of GFAP and EAAT2 was validated by western blots (Supplementary Figure 3, *N* = 1), confirming gene expression analysis seen by RT qPCR. These results suggest that acute serum exposure induces morphological changes in astrocytes in a dose-dependent manner. However, acute exposure to serum seems to activate different signaling pathways in astrocytes compared to chronic serum-culture condition, as astrocyte and inflammatory markers were expressed differently in these conditions.

### EVs derived from serum-cultured astrocytes have a smaller size and can induce astrocyte reactivity

To investigate whether chronic serum culture affects the morphology and protein cargoes of ADEVs, we optimized an isolation procedure to effectively collect a large quantity of ADEVs from serum-free astrocytes. In brief, conditioned medium collection was completed four times from a 6-well dish culture until 48 ml of conditioned medium was collected. For the serum-culture condition, astrocytes were initially seeded into four T75 flasks with serum-containing medium for 24 h before medium was replaced with astrocyte medium without FBS. Cells were then cultured for 72 h before conditioned medium was collected.

**Figure 6:**
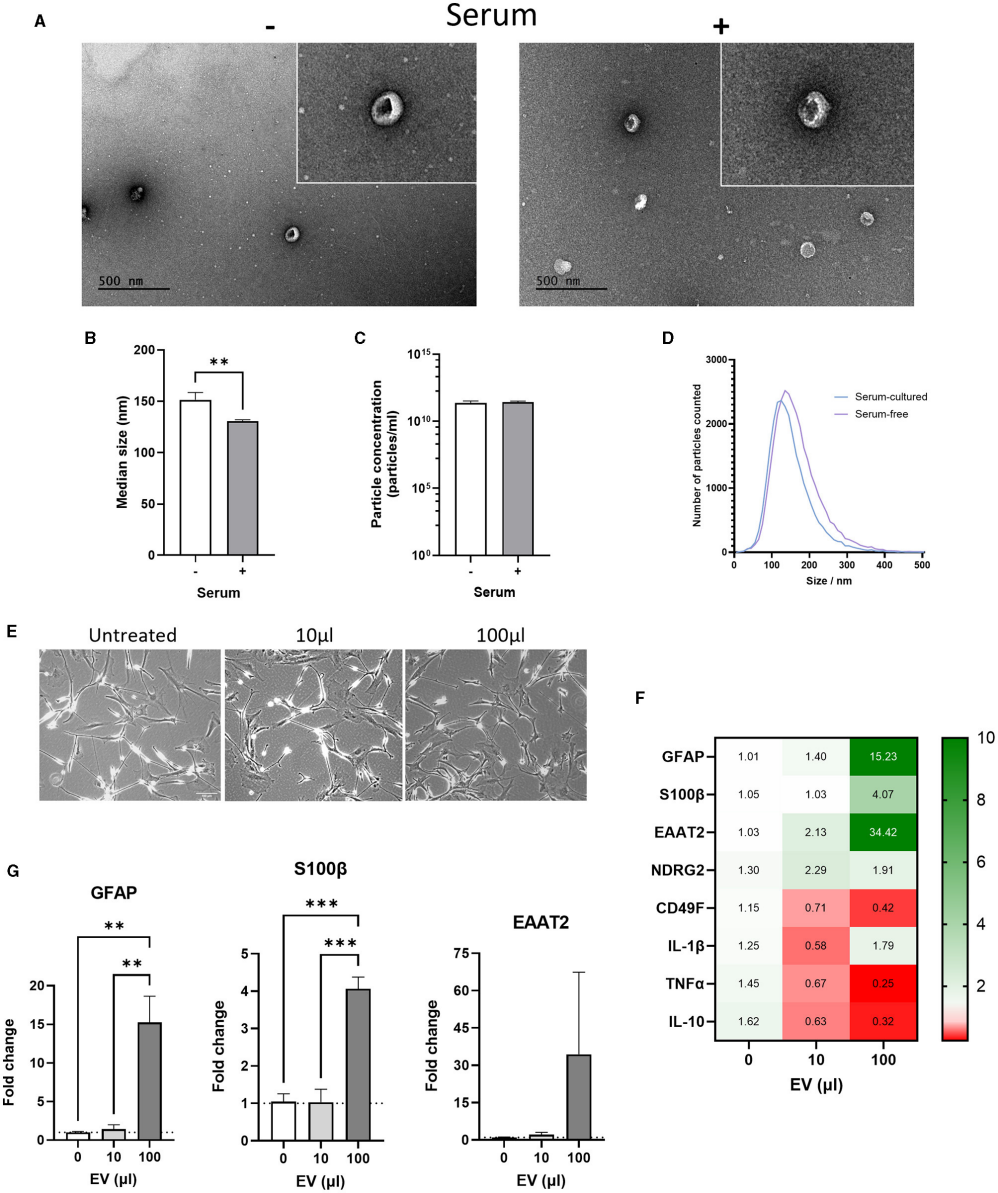
Comparison of serum-free and serum-cultured astrocyte-derived extracellular vesicles (ADEVs). **(A)** Transmission electron microscopy (TEM) images showing cup-shaped serum-free and serum-cultured ADEVs. **(B)** Nanoparticle tracking analysis (NTA) showing a significant increase in the median size of serum-free ADEVs compared to serum-cultured ADEVs (**p < 0.01, *N* = 7–9). **(C)** No difference in particle concentration was identified using NTA between ADEVs (mean = 2 × 10^11^ particles/mL, *N* = 7–9). **(D)** Size distribution of ADEVs showing a similar size range of in both serum-free and serum-cultured ADEVs (between 50 and 300 nm diameter). However, a shift toward larger EVs was observed in serum-free ADEVs. **(E)** Representative brightfield images of serum-free astrocytes treated with either 10 μL or 100 μL of serum-cultured ADEVs (2 × 10^11^ particles/mL). **(F)** Heatmap showing that serum-cultured ADEVs dose-dependently altered gene expression of astrocyte and inflammatory cytokine markers in serum-free astrocytes. **(G)** Real-time qPCR analysis showing a significant increase in GFAP and S100β gene expression after treatment with 100 μL of serum-cultured ADEVs, with an increasing trend also observed in EAAT2 gene expression (**p < 0.01, ***p < 0.001, *N* = 3). Data represent mean ± SEM and were analyzed using unpaired *t*-test **(B, C)** or one-way ANOVAs followed by Dunnett's multiple comparisons test **(G)**. Measurements were performed after 1 week in culture.

ADEVs were isolated from conditioned medium using a combination of ultrafiltration and size exclusion chromatography (SEC) resulting in three EV-enriched fractions. Transmission Electron Microscopy (TEM) was first used on combined EV-enriched fractions to confirm the presence of EV-like particles. Both lipid membrane and characteristic cup-shaped morphology were evident under vacuum in EV-enriched fractions from serum-free and serum-cultured astrocytes (Figure 6A). Nanoparticle tracking analysis (NTA) was then used to measure size and concentration of particles in EV-enriched fractions. Serum-cultured ADEVs were significantly smaller than those released from serum-free astrocytes with a median size of 130.6 ± 1.5 nm for serum-cultured ADEVs compared to 151.3 ± 7.1 nm for serum-free ADEVs (Figure 6B, *p* < 0.001, *N* = 7–9). No difference in concentration of particles was detected, suggesting ADEV isolation was comparable for both serum and serum-free conditioned media (Figure 6C). Size distribution was similar between serum-cultured and serum free groups with particles having a diameter ranging from 100 to 200 nm (Figure 6D).

Next, we investigated whether ADEVs isolated from serum-cultured astrocytes could have a functional effect on serum-free astrocytes. To this end, we treated serum-free astrocytes with either 10 μL (2 × 10^9^ particles) or 100 μL (2 × 10^10^ particles) of EVs-derived from serum-cultured astrocytes for 24 h. No morphological change was evident in serum-free astrocytes, with area/perimeter ratios remaining the same after EV treatment (Figure 6E, *N* = 3). For the 10 μL condition, there was no significant change in gene and protein expression of astrocyte and inflammatory markers (Figures 6F, G and Supplementary Figure 4). Interestingly, there was a significant increase in the expression of astrocyte markers for the 100 μL condition, whilst inflammatory markers were either downregulated or not affected (Figures 6F, G, *p* < 0.01–0.001, *N* = 3). Then, the effect of EVs-derived from serum-cultured astrocytes on cellular stress response such as the unfolded protein response (UPR) was investigated by assessing the expression of HSP70. In this case, there was no significant change in gene expression of HSP70 for both 10 and 100 μL EV treatments (Supplementary Figure 5A, *N* = 3) and no evident change in protein levels by western-blot analysis (Supplementary Figure 5B, *N* = 1).

This indicates that EVs may transport bioactive molecules capable of propagating a reactive astrocyte phenotype through mechanisms distinct from those involved in both inflammatory processes and UPR.

### Proteomic characterization of ADEVs

**Figure 7:**
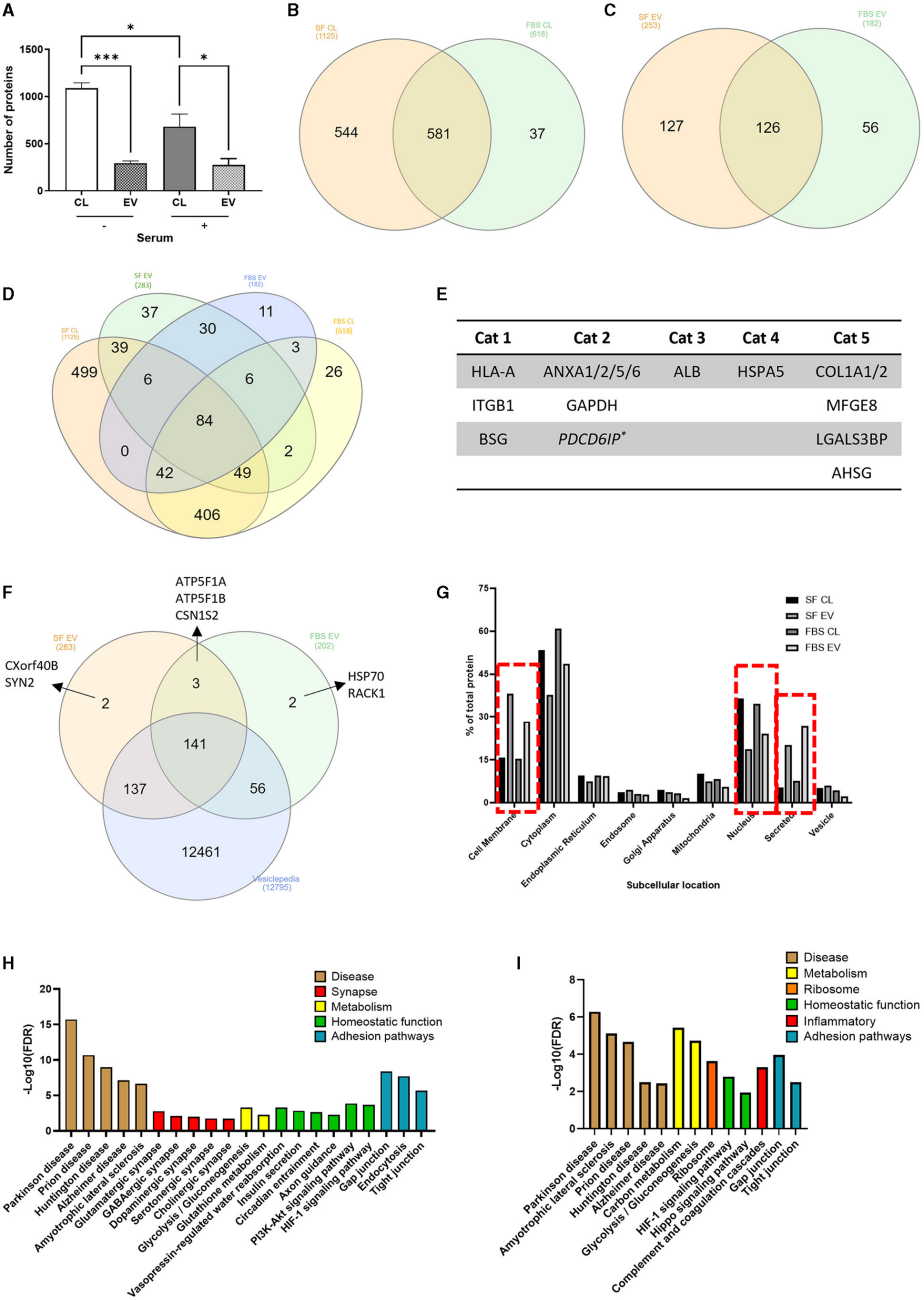
Proteomic analysis of serum-free and serum-cultured astrocytes and their ADEVs. **(A)** The number of unique proteins identified in serum-free and serum-cultured astrocyte cell lysates and their EVs (*p < 0.05, ***p < 0.001, *N* = 3). Half of the total proteins identified within **(B)** the cell lysates and **(C)** ADEVs were detected in both serum-free and serum-cultured conditions. **(D)** Venn diagram displaying the overlap of proteins found within all 4 conditions. **(E)** Identification of EV associated proteins (Category 1 and 2) as well as a lack of contaminant (Category 3) or cell-based (Category 4) proteins, as described in the MISEV guidelines. Secreted proteins were also commonly found within ADEV samples (Category 5). **(F)** The majority of ADEV proteins were also found within the Vesiclepedia database. **(G)** Subcellular location of the detected proteins highlighting an increase in cell membrane and secreted proteins within ADEV samples and an increase in nuclear proteins within cell lysate samples. **(H)** KEGG pathway analysis of serum-free ADEVs proteomes highlighting many synapse-related pathways such as glutamatergic and GABAergic synapse pathways, as well as many homeostatic pathways such as insulin secretion and axon guidance. **(I)** KEGG pathway analysis of serum cultured ADEVs showing disease pathways such as Parkinson's disease and ALS, as well as an enrichment in complement and coagulation cascades. Data represent mean ± SEM and were analyzed using one-way ANOVA followed by Tukey's multiple comparison test. Measurements were performed after 1 week in culture.

To determine the impact of serum-induced reactivity at the level of the astrocyte whole cell and ADEV proteomes, an exploratory analysis was performed using LC-MS/MS. Based on protein identification in at least 2/3 repeats, significantly more proteins were detected in serum-free cell lysates, with an average of 1,088 proteins detected compared to 680 proteins identified in the serum-cultured cell lysates (Figure 7A, *p* < 0.05–0.001, *N* = 3).

After manual filtering of proteomic data for common contaminants such as keratin and albumin, 1,162 proteins were detected in whole-cell lysates across serum-free and serum-cultured astrocytes, and 309 proteins in the corresponding purified ADEVs. Comparing simple presence and absence of protein detection (indicative of major protein expression differences) in the astrocyte whole-cell lysates of serum-free and serum-cultured cells, we noted that only 581 (50%) of all proteins detected were common to both conditions, with 543 (47%) of all proteins detected only in the serum-free cells, and 37 (3%) of all proteins detected only in the serum-cultured cells (Figure 7B), indicative of overlapping but potentially distinct proteomes. Indeed, when comparing the corresponding EV proteomes, 126 proteins (40%) of all EV proteins detected were found to be common between serum-free and serum cultured ADEVs, with 127 (41%) of all proteins detected exclusively in the serum-free ADEVs and 56 (18%) of proteins exclusive to the serum-cultured ADEVs (Figure 7C), again indicating distinct sub-proteomes. When comparing the ADEVs and their corresponding whole-cell lysates, 30 (of 126) proteins were detected within both ADEV samples but not the parent cell lysates (Figure 7D), suggesting a selective enrichment of these proteins in EVs. Key proteins as described in the MISEV (Minimal Information for Studies of Extracellular Vesicles) guidelines (Welsh et al., 2024) were detected within purified EV samples (Figure 7E). The majority of ADEV proteins were found within the Vesiclepedia database (Figure 7F), a comprehensive collection of previously identified proteins found with EV samples from published studies (Kalra et al., 2012). Cellular localization of proteins detected with all the samples (cell lysates and EVs) using KEGG analysis highlighted an enrichment of “cell membrane” proteins and “secreted proteins” within the ADEV samples compared to an enrichment of “nuclear proteins” within the cell lysates, further supporting selective protein loading into ADEV (Figure 7G).

KEGG pathway analysis of proteins within the ADEV proteomes also indicated an enrichment in “synapse-related” and “homeostatic” pathways in EVs from quiescent astrocytes, suggesting (were such proteins transferrable to neurons) a potentially neuro-supportive cellular phenotype (Figure 7H). These pathways were not enriched in EV proteomes from the reactive astrocytes, with “complement” and “coagulation cascades” found to be enriched pathway instead (Figure 7I). Overall, our proteome analyses confirm that primary human astrocytes have a remodeled proteomic phenotype in response to chronic culture with serum, consistent with a shift toward a reactive phenotype.

## Discussion

The nature of currently incurable human CNS diseases like Alzheimer's disease or amyotrophic lateral sclerosis (ALS) with pathological astrocyte components underscores the necessity of human cell-based models alongside traditional animal models for unraveling the role of ADEVs in pathological mechanisms (Varcianna et al., 2019; You et al., 2020) and advancing the development of effective treatments for CNS pathologies (Cai et al., 2017; Barbar et al., 2020; Guttenplan et al., 2020).

Here, we demonstrate that culturing human primary astrocytes in a serum-free medium recapitulates *in vivo* phenotypic traits, as evidenced by morphological and transcriptional analysis. Prolonged serum exposure diminishes astrocyte marker expression, leading to irreversible cell stress, while short-term exposure mirrors previous findings on astrocyte reactivity, suggesting distinct signaling pathways for acute and chronic reactivity. We show that chronically serum-cultured astrocytes secrete smaller ADEVs than serum-free astrocytes and can propagate their reactive phenotype to quiescent cells. Each type of ADEVs exhibits unique protein profiles, offering insights into ADEVs as potential biomarkers of CNS pathologies.

Serum-free medium maintains primary astrocytes in resting state and promotes cell proliferation when supplemented with vitamins, growth factors and hormones (Morrison and De Vellis, 1983). In line with previous works, we show that human primary fetal astrocytes from commercial sources can be maintained in a healthy and resting state for several weeks using advanced-DMEM and G5 supplements. However, serum-free human astrocytes did not appear to proliferate *in vitro*, even with addition of EGF and FGF, contrasting with previous studies (Foo et al., 2011; Zhang et al., 2016; Prah et al., 2019), and suggesting a potentially more quiescent phenotype in our model. Consistently, our findings indicate that serum-free human primary astrocytes closely resemble the morphology of *in vivo* astrocytes. In contrast, chronically serum-cultured astrocytes display an irreversible fibroblast-like morphology characterized by enlarged nuclei (McCarthy and De Vellis, 1980; Foo et al., 2011; Zhang et al., 2016; Prah et al., 2019). Importantly, these morphological changes occur rapidly (within < 3 h), are dependent on the FBS dose and more pronounced than those induced by cytokine stimulation (TNFα, IL-1α, and C1q) (Liddelow et al., 2017). Additionally, these morphological changes appear to be unaffected by the duration of serum exposure (when comparing short vs. long-term exposure). While indicative of astrocyte reactivity, we hypothesize that the morphological alterations induced by serum cannot be solely attributed to an inflammatory response but rather signify a physiological stress stemming from cell proliferation (Walz and Lang, 1998; Xu, 2018; Cibelli et al., 2021).

Our transcriptional analysis reveals that human primary astrocytes exist in two distinct reactive states in serum condition: a chronic state associated with maturation and inflammatory senescence and an acute state characteristic of reactive astrocytes cultured *in vitro* (Prah et al., 2019). While GFAP is a well-established marker of astrocyte reactivity (Sofroniew and Vinters, 2010; Prah et al., 2019; Escartin et al., 2021), our findings do not correlate with increased GFAP expression in chronically serum-cultured astrocytes (Du et al., 2010; Prah et al., 2019). Notably, the expression of well-characterized cell-specific genes for astrocytes (GFAP, S100β and EAAT2) is at least three-fold higher in serum-free astrocytes compared to serum-cultured astrocytes. It is not uncommon for GFAP expression to be downregulated after passage of astrocytes in FBS-containing medium (Xu, 2018; Cibelli et al., 2021). Additionally, astrocytes are heterogeneous in nature (Zamanian et al., 2012) and subpopulations of astrocytes exist in culture (Walz and Lang, 1998; Tatsumi et al., 2018; Barbar et al., 2020). We therefore speculate that the reduced levels of astrocyte markers in serum condition may be explained by their high proliferative profile, in which cells may invest more in the synthesis of proteins related to cell proliferation instead of specific astrocytic markers. This hypothesis is supported by the observed enlargement of nuclei and elevated levels of IL-1β and IL-10 in serum-cultured astrocytes, characteristic of excessive protein production and an inflammatory secretory phenotype, respectively.

Interestingly, when serum-free astrocytes are cultured in FBS-containing medium for < 24 h (i.e., acute response), we report elevated levels of GFAP in these cells, aligning with previous findings (Du et al., 2010; Prah et al., 2019). Of all the astrocyte markers examined, we have determined that GFAP exhibits the highest sensitivity as a marker of acute astrocyte response compared to novel markers of human astrocyte reactivity, including CD49F (Barbar et al., 2020) and NDRG2 (Flugge et al., 2014). Furthermore, serum-free astrocytes did not acquire an A1-like reactive phenotype upon stimulation with TNFα, IL-1α, and C1q (Liddelow et al., 2017; Barbar et al., 2020), despite showing evident production of pro-inflammatory cytokines. Consequently, we are unable to provide insights on inflammatory A1 reactive astrocytes and their potential neurotoxic role (Liddelow et al., 2017; Barbar et al., 2020).

We advocate for further investigation into the acute reactive state of astrocytes, enabling comparisons with the effects of cytokines (Liddelow et al., 2017; Barbar et al., 2020), growth factors (Prah et al., 2019) and FBS-containing medium (Du et al., 2010; Foo et al., 2011; Zhang et al., 2016; Prah et al., 2019) on astrocytes. Additionally, more emphasis should be made on multiple molecular and functional parameters, ideally *in vivo*, to assess reactive astrocytes instead of relying on a single marker (e.g., GFAP) (Escartin et al., 2021). Finally, our RNA-sequencing analysis reveals that serum-free astrocytes retain *in vivo* phenotypic traits, whilst serum-cultured astrocytes exhibit diminished function related to synapse formation and neurotransmitter uptake, together with increased cell proliferation, inflammatory senescence, and pathways related to CNS diseases. This represents an initial step toward translating astrocyte reactivity *in vitro* across various CNS pathological contexts (Zamanian et al., 2012; Zhang et al., 2016). Nevertheless, to validate these molecular profiles effectively, further investigations necessitate functional validation *in vivo*.

The morphological characterization of EVs reveals a diverse population of ADEVs, which varies depending on the phenotypic state of astrocytes. Specifically, smaller ADEVs are observed in chronically serum-cultured (reactive) astrocytes compared to serum-free (resting) astrocytes. This has not been observed before in human and rodents ADEVs, even after cytokine treatments (Chaudhuri et al., 2018; Datta Chaudhuri et al., 2020; You et al., 2020), confirming that the effect of FBS-containing medium on astrocyte reactivity surpasses that of inflammatory stimuli and could potentially occur independently. There are different subtypes of EVs including exosomes (30–200 nm) and microvesicles (150–1000 nm) (Couch et al., 2021), which have been shown to be released through different mechanisms depending on the physiological state of the cells (You et al., 2020). For instance, a form of small EV (median size ~80 nm) promotes cell proliferation and may therefore be released in serum-cultured astrocytes but not in serum-free astrocytes, reducing the overall median size of ADEVs from reactive astrocytes (Lee et al., 2019).

The proteome analysis of ADEVs and their parent cells confirms that ADEV proteome is not a replica of the parent proteome but rather suggests a selective loading mechanism wherein specific proteins are preferentially incorporated into EVs. Furthermore, protein differences were identified between quiescent and reactive ADEVs, supporting previous proteomic analysis of reactive ADEVs (Datta Chaudhuri et al., 2020; You et al., 2020). Pathway analysis highlighted neuroprotective and homeostatic pathways in serum-free ADEVs, contrasting with serum-cultured astrocytes, where ribosomal pathways are more prominent, along with complement and coagulation cascades known to be associated with CNS pathologies (Kjaeldgaard et al., 2018). Again, this represents an initial step toward translating ADEVs as biomarkers of CNS pathologies, and a deeper proteomic analysis is needed to confirm these data. Given the challenges associated with using EVs as biomarkers [see review by Couch et al. (2021)], we also recommend that more emphasis should be made on multiple structural, molecular and functional parameters, ideally measured *in vivo*, to validate ADEVs in biological fluids as biomarkers of CNS pathologies.

The reactive astrocyte secretome has been identified as potentially neurotoxic (Liddelow et al., 2017; Guttenplan et al., 2020). Given the changes observed in ADEVs during reactivity, we hypothesize that reactive ADEVs could be involved in the neurotoxicity exhibited by astrocytes in pathology (Silverman et al., 2019). The effect of serum-cultured ADEVs on the upregulation of astrocyte markers (GFAP and S100β) in resting astrocytes partially supports this hypothesis. We further speculate that ADEVs may play a role in a mechanism that propagates the neuroprotective and/or neurotoxic effects of reactive astrocytes (Varcianna et al., 2019; Datta Chaudhuri et al., 2020), but this warrants further functional investigation.

With regard to the limitations of this study, we did not consider metabolic and functional alterations in serum-cultured astrocytes, which are essential for fully characterizing the impact of serum on commercially available human astrocytes (Zhang et al., 2016; Prah et al., 2019). Furthermore, we did not investigate changes in signaling pathways associated with reactive astrocytes (e.g., JAK/STAT3 vs. NFκB pathways) (Ben Haim et al., 2015; Sarmiento Soto et al., 2020), nor did we investigate the mechanism by which serum cultured ADEVs mimics acute astrocyte reactivity. Complete EV depletion of serum is currently impossible, and studies have shown that EV-depleted serum can alter cell behavior and growth (Lehrich et al., 2018). The concentration of proteins present in serum-cultured medium could potentially trigger astrocyte reactivity to ADEVs and therefore requires further functional investigation. Nevertheless, transitioning toward serum-free cultures would eliminate this issue in ADEV studies.

In summary, this study demonstrates that human primary astrocytes cultured in serum-free medium are akin to *in vivo* resting astrocytes and maintaining these cells in serum should be avoided as it induces permanent changes that are specific to human reactive astrocytes. These changes are also reflected in their ADEVs suggesting that markers of human primary astrocytes need to be evaluated using multiple molecular and functional parameters before using their ADEVs in functional studies or as predictive biomarkers of astrocyte health.

## Data availability statement

The original datasets presented in this study can be found here: https://data.mendeley.com/datasets/ms94f4hz35/1.

## Ethics statement

Ethical approval was not required for the studies on humans in accordance with the local legislation and institutional requirements because only commercially available established cell lines were used.

## Author contributions

KW: Supervision, Funding acquisition, Writing – review & editing, Writing – original draft, Visualization, Validation, Software, Resources, Project administration, Methodology, Investigation, Formal analysis, Data curation, Conceptualization. HB: Writing – review & editing, Investigation, Data curation, Conceptualization. BS: Writing – review & editing, Methodology, Investigation, Data curation. PG: Writing – review & editing, Software, Formal analysis. RM-R: Writing – review & editing, Methodology. DS: Writing – review & editing, Conceptualization. RL: Writing – review & editing, Writing – original draft, Resources, Methodology, Formal analysis, Conceptualization. SS: Writing – review & editing, Writing – original draft, Visualization, Validation, Supervision, Software, Resources, Project administration, Methodology, Investigation, Funding acquisition, Formal analysis, Data curation, Conceptualization.

## References

[B1] Andersen J. V. Schousboe A. (2023). Milestone review: metabolic dynamics of glutamate and GABA mediated neurotransmission - The essential roles of astrocytes. J. Neurochem. 166, 109–137. 10.1111/jnc.15811 36919769

[B2] Arredondo C. Cefaliello C. Dyrda A. Jury N. Martinez P. Diaz I.. (2022). Excessive release of inorganic polyphosphate by ALS/FTD astrocytes causes non-cell-autonomous toxicity to motoneurons. Neuron 110, 1656–1670. 10.1016/j.neuron.2022.02.010 35276083 PMC9119918

[B3] Aswad H. Jalabert A. Rome S. (2016). Depleting extracellular vesicles from fetal bovine serum alters proliferation and differentiation of skeletal muscle cells in vitro. BMC Biotechnol. 16:32. 10.1186/s12896-016-0262-0 27038912 PMC4818850

[B4] Barbar L. Jain T. Zimmer M. Kruglikov I. Sadick J. S. Wang M.. (2020). CD49f is a novel marker of functional and reactive human iPSC-derived astrocytes. Neuron 107, 436–453 e412. 10.1016/j.neuron.2020.05.014 32485136 PMC8274549

[B5] Ben Haim L. Ceyzeriat K. Carrillo-De Sauvage M. A. Aubry F. Auregan G. Guillermier M.. (2015). The JAK/STAT3 pathway is a common inducer of astrocyte reactivity in Alzheimer's and Huntington's diseases. J. Neurosci. 35, 2817–2829. 10.1523/JNEUROSCI.3516-14.2015 25673868 PMC6605603

[B6] Brandebura A. N. Paumier A. Onur T. S. Allen N. J. (2023). Astrocyte contribution to dysfunction, risk and progression in neurodegenerative disorders. Nat. Rev. Neurosci. 24, 23–39. 10.1038/s41583-022-00641-1 36316501 PMC10198620

[B7] Broadhurst D. Goodacre R. Reinke S. N. Kuligowski J. Wilson I. D. Lewis M. R.. (2018). Guidelines and considerations for the use of system suitability and quality control samples in mass spectrometry assays applied in untargeted clinical metabolomic studies. Metabolomics 14:72. 10.1007/s11306-018-1367-3 29805336 PMC5960010

[B8] Cai Z. Wan C. Q. Liu Z. (2017). Astrocyte and Alzheimer's disease. J. Neurol. 264, 2068–2074. 10.1007/s00415-017-8593-x 28821953

[B9] Chaudhuri A. D. Dastgheyb R. M. Yoo S. W. Trout A. Talbot C. C. Jr. Hao H.. (2018). TNFalpha and IL-1beta modify the miRNA cargo of astrocyte shed extracellular vesicles to regulate neurotrophic signaling in neurons. Cell Death Dis. 9:363. 10.1038/s41419-018-0369-4 29507357 PMC5838212

[B10] Cibelli A. Veronica Lopez-Quintero S. Mccutcheon S. Scemes E. Spray D. C. Stout R. F. Jr.. (2021). Generation and characterization of immortalized mouse cortical astrocytes from wildtype and connexin43 knockout mice. Front. Cell Neurosci. 15:647109. 10.3389/fncel.2021.647109 33790744 PMC8005635

[B11] Codeluppi S. Gregory E. N. Kjell J. Wigerblad G. Olson L. Svensson C. I. (2011). Influence of rat substrain and growth conditions on the characteristics of primary cultures of adult rat spinal cord astrocytes. J. Neurosci Methods. 197, 118–127. 10.1016/j.jneumeth.2011.02.011 21345349

[B12] Couch Y. Buzas E. I. Di Vizio D. Gho Y. S. Harrison P. Hill A. F.. (2021). A brief history of nearly EV-erything - The rise and rise of extracellular vesicles. J. Extracell Ves. 10:e12144. 10.1002/jev2.12144 34919343 PMC8681215

[B13] Datta Chaudhuri A. Dasgheyb R. M. Devine L. R. Bi H. Cole R. N. Haughey N. J. (2020). Stimulus-dependent modifications in astrocyte-derived extracellular vesicle cargo regulate neuronal excitability. Glia 68, 128–144. 10.1002/glia.23708 31469478

[B14] Dickens A. M. Tovar Y. R. L. B. Yoo S. W. Trout A. L. Bae M. Kanmogne M.. (2017). Astrocyte-shed extracellular vesicles regulate the peripheral leukocyte response to inflammatory brain lesions. Sci Signal 10:7696. 10.1126/scisignal.aai7696 28377412 PMC5590230

[B15] Du F. Qian Z. M. Zhu L. Wu X. M. Qian C. Chan R.. (2010). Purity, cell viability, expression of GFAP and bystin in astrocytes cultured by different procedures. J. Cell Biochem. 109, 30–37. 10.1002/jcb.22375 19899109

[B16] Escartin C. Galea E. Lakatos A. O'callaghan J. P. Petzold G. C. Serrano-Pozo A.. (2021). Reactive astrocyte nomenclature, definitions, and future directions. Nat. Neurosci. 24, 312–325. 10.1038/s41593-020-00783-4 33589835 PMC8007081

[B17] Escartin C. Guillemaud O. Carrillo-De Sauvage M. A. (2019). Questions and (some) answers on reactive astrocytes. Glia 67, 2221–2247. 10.1002/glia.23687 31429127

[B18] Flugge G. Araya-Callis C. Garea-Rodriguez E. Stadelmann-Nessler C. Fuchs E. (2014). NDRG2 as a marker protein for brain astrocytes. Cell Tissue Res. 357, 31–41. 10.1007/s00441-014-1837-5 24816982 PMC4077251

[B19] Foo L. C. Allen N. J. Bushong E. A. Ventura P. B. Chung W. S. Zhou L.. (2011). Development of a method for the purification and culture of rodent astrocytes. Neuron 71, 799–811. 10.1016/j.neuron.2011.07.022 21903074 PMC3172573

[B20] Guttenplan K. A. Weigel M. K. Adler D. I. Couthouis J. Liddelow S. A. Gitler A. D.. (2020). Knockout of reactive astrocyte activating factors slows disease progression in an ALS mouse model. Nat. Commun. 11:3753. 10.1038/s41467-020-17514-9 32719333 PMC7385161

[B21] Guttenplan K. A. Weigel M. K. Prakash P. Wijewardhane P. R. Hasel P. Rufen-Blanchette U.. (2021). Neurotoxic reactive astrocytes induce cell death via saturated lipids. Nature 599, 102–107. 10.1038/s41586-021-03960-y 34616039 PMC12054010

[B22] Hart T. Komori H. K. Lamere S. Podshivalova K. Salomon D. R. (2013). Finding the active genes in deep RNA-seq gene expression studies. BMC Genom. 14:778. 10.1186/1471-2164-14-778 24215113 PMC3870982

[B23] Heckenbach I. Mkrtchyan G. V. Ezra M. B. Bakula D. Madsen J. S. Nielsen M. H.. (2022). Nuclear morphology is a deep learning biomarker of cellular senescence. Nat. Aging 2, 742–755. 10.1038/s43587-022-00263-3 37118134 PMC10154217

[B24] Kalra H. Simpson R. J. Ji H. Aikawa E. Altevogt P. Askenase P.. (2012). Vesiclepedia: a compendium for extracellular vesicles with continuous community annotation. PLoS Biol. 10:e1001450. 10.1371/journal.pbio.1001450 23271954 PMC3525526

[B25] Kjaeldgaard A. L. Pilely K. Olsen K. S. Pedersen S. W. Lauritsen A. O. Moller K.. (2018). Amyotrophic lateral sclerosis: the complement and inflammatory hypothesis. Mol. Immunol. 102, 14–25. 10.1016/j.molimm.2018.06.007 29933890

[B26] Lee S. S. Won J. H. Lim G. J. Han J. Lee J. Y. Cho K. O.. (2019). A novel population of extracellular vesicles smaller than exosomes promotes cell proliferation. Cell Commun Signal 17:95. 10.1186/s12964-019-0401-z 31416445 PMC6694590

[B27] Lehrich B. M. Liang Y. Khosravi P. Federoff H. J. Fiandaca M. S. (2018). Fetal bovine serum-derived extracellular vesicles persist within vesicle-depleted culture media. Int. J. Mol. Sci. 19:538. 10.3390/ijms19113538 30423996 PMC6275013

[B28] Lian H. Yang L. Cole A. Sun L. Chiang A. C. Fowler S. W.. (2015). NFkappaB-activated astroglial release of complement C3 compromises neuronal morphology and function associated with Alzheimer's disease. Neuron 85, 101–115. 10.1016/j.neuron.2014.11.018 25533482 PMC4289109

[B29] Liddelow S. A. Barres B. A. (2017). Reactive astrocytes: production, function, and therapeutic potential. Immunity 46, 957–967. 10.1016/j.immuni.2017.06.006 28636962

[B30] Liddelow S. A. Guttenplan K. A. Clarke L. E. Bennett F. C. Bohlen C. J. Schirmer L.. (2017). Neurotoxic reactive astrocytes are induced by activated microglia. Nature 541, 481–487. 10.1038/nature21029 28099414 PMC5404890

[B31] Love M. I. Huber W. Anders S. (2014). Moderated estimation of fold change and dispersion for RNA-seq data with DESeq2. Genome Biol. 15:550. 10.1186/s13059-014-0550-8 25516281 PMC4302049

[B32] McCarthy K. D. De Vellis J. (1980). Preparation of separate astroglial and oligodendroglial cell cultures from rat cerebral tissue. J. Cell Biol. 85, 890–902. 10.1083/jcb.85.3.890 6248568 PMC2111442

[B33] Mellacheruvu D. Wright Z. Couzens A. L. Lambert J. P. St-Denis N. A. Li T.. (2013). The CRAPome: a contaminant repository for affinity purification-mass spectrometry data. Nat. Methods 10, 730–736. 10.1038/nmeth.2557 23921808 PMC3773500

[B34] Morrison R. S. De Vellis J. (1983). Differentiation of purified astrocytes in a chemically defined medium. Brain Res. 285, 337–345. 10.1016/0165-3806(83)90030-5 6354363

[B35] Oberheim N. A. Takano T. Han X. He W. Lin J. H. Wang F.. (2009). Uniquely hominid features of adult human astrocytes. J. Neurosci. 29, 3276–3287. 10.1523/JNEUROSCI.4707-08.2009 19279265 PMC2819812

[B36] Oberheim N. A. Wang X. Goldman S. Nedergaard M. (2006). Astrocytic complexity distinguishes the human brain. Trends Neurosci. 29, 547–553. 10.1016/j.tins.2006.08.004 16938356

[B37] Prah J. Winters A. Chaudhari K. Hersh J. Liu R. Yang S. H. (2019). A novel serum free primary astrocyte culture method that mimic quiescent astrocyte phenotype. J. Neurosci. Methods 320, 50–63. 10.1016/j.jneumeth.2019.03.013 30904500 PMC6546087

[B38] Rouillard M. E. Sutter P. A. Durham O. R. Willis C. M. Crocker S. J. (2021). Astrocyte-derived extracellular vesicles (ADEVs): deciphering their influences in aging. Aging Dis. 12, 1462–1475. 10.14336/AD.2021.0608 34527422 PMC8407882

[B39] Salek R. M. Xia J. Innes A. Sweatman B. C. Adalbert R. Randle S.. (2010). A metabolomic study of the CRND8 transgenic mouse model of Alzheimer's disease. Neurochem. Int. 56, 937–947. 10.1016/j.neuint.2010.04.001 20398713

[B40] Sarmiento Soto M. Larkin J. R. Martin C. Khrapitchev A. A. Maczka M. Economopoulos V.. (2020). STAT3-mediated astrocyte reactivity associated with brain metastasis contributes to neurovascular dysfunction. Cancer Res. 12:2251. 10.1158/0008-5472.CAN-20-2251 33106335

[B41] Silverman J. M. Christy D. Shyu C. C. Moon K. M. Fernando S. Gidden Z.. (2019). CNS-derived extracellular vesicles from superoxide dismutase 1 (SOD1)(G93A) ALS mice originate from astrocytes and neurons and carry misfolded SOD1. J. Biol. Chem. 294, 3744–3759. 10.1074/jbc.RA118.004825 30635404 PMC6416428

[B42] Sofroniew M. V. Vinters H. V. (2010). Astrocytes: biology and pathology. Acta. Neuropathol. 119, 7–35. 10.1007/s00401-009-0619-8 20012068 PMC2799634

[B43] Soto M. S. O'brien E. R. Andreou K. Scrace S. F. Zakaria R. Jenkinson M. D.. (2016). Disruption of tumour-host communication by downregulation of LFA-1 reduces COX-2 and e-NOS expression and inhibits brain metastasis growth. Oncotarget 7, 52375–52391. 27447568 10.18632/oncotarget.10737 PMC5239559

[B44] Stuendl A. Kraus T. Chatterjee M. Zapke B. Sadowski B. Moebius W.. (2021). Alpha-synuclein in plasma-derived extracellular vesicles is a potential biomarker of Parkinson's disease. Mov. Disord. 36, 2508–2518. 10.1002/mds.28639 34002893

[B45] Tatsumi K. Isonishi A. Yamasaki M. Kawabe Y. Morita-Takemura S. Nakahara K.. (2018). Olig2-lineage astrocytes: a distinct subtype of astrocytes that differs from GFAP astrocytes. Front. Neuroanat. 12:8. 10.3389/fnana.2018.00008 29497365 PMC5819569

[B46] The UniProt Consortium (2021). UniProt: the universal protein knowledgebase in 2021. Nucleic Acids Res. 49, D480–D489. 10.1093/nar/gkaa1100 33237286 PMC7778908

[B47] Varcianna A. Myszczynska M. A. Castelli L. M. O'neill B. Kim Y. Talbot J.. (2019). Micro-RNAs secreted through astrocyte-derived extracellular vesicles cause neuronal network degeneration in C9orf72 ALS. EBioMedicine 40, 626–635. 10.1016/j.ebiom.2018.11.067 30711519 PMC6413467

[B48] Walz W. Lang M. K. (1998). Immunocytochemical evidence for a distinct GFAP-negative subpopulation of astrocytes in the adult rat hippocampus. Neurosci. Lett. 257, 127–130. 10.1016/S0304-3940(98)00813-1 9870336

[B49] Welsh J. A. Goberdhan D. C. I. O'driscoll L. Buzas E. I. Blenkiron C. Bussolati B.. (2024). Minimal information for studies of extracellular vesicles (MISEV2023): From basic to advanced approaches. J. Extracell Vesicles 13:e12404. 10.1002/jev2.12404 38326288 PMC10850029

[B50] Willis C. M. Menoret A. Jellison E. R. Nicaise A. M. Vella A. T. Crocker S. J. (2017). A refined bead-free method to identify astrocytic exosomes in primary glial cultures and blood plasma. Front. Neurosci. 11:335. 10.3389/fnins.2017.00335 28663721 PMC5471332

[B51] Xu J. (2018). New insights into GFAP negative astrocytes in calbindin D28k immunoreactive astrocytes. Brain Sci. 8:143. 10.3390/brainsci8080143 30081456 PMC6119979

[B52] You Y. Borgmann K. Edara V. V. Stacy S. Ghorpade A. Ikezu T. (2020). Activated human astrocyte-derived extracellular vesicles modulate neuronal uptake, differentiation and firing. J. Extracell Vesicles 9:1706801. 10.1080/20013078.2019.1706801 32002171 PMC6968484

[B53] Zamanian J. L. Xu L. Foo L. C. Nouri N. Zhou L. Giffard R. G.. (2012). Genomic analysis of reactive astrogliosis. J. Neurosci. 32, 6391–6410. 10.1523/JNEUROSCI.6221-11.2012 22553043 PMC3480225

[B54] Zhang Y. Sloan S. A. Clarke L. E. Caneda C. Plaza C. A. Blumenthal P. D.. (2016). Purification and characterization of progenitor and mature human astrocytes reveals transcriptional and functional differences with mouse. Neuron 89, 37–53. 10.1016/j.neuron.2015.11.013 26687838 PMC4707064

[B55] Zhao S. Sheng S. Wang Y. Ding L. Xu X. Xia X.. (2021). Astrocyte-derived extracellular vesicles: a double-edged sword in central nervous system disorders. Neurosci. Biobehav. Rev. 125, 148–159. 10.1016/j.neubiorev.2021.02.027 33626395

