# Supplementary material for: A convenient model of serum-induced reactivity of human astrocytes to investigate astrocyte-derived extracellular vesicles
Source: Front Cell Neurosci. 2024 Jun 10;18:1414142. doi: 10.3389/fncel.2024.1414142 (PMC11195030; doi:10.3389/fncel.2024.1414142)
Supplement: Supplementary file 1 [file Data_Sheet_1.docx]

**A convenient model of serum-induced reactivity of human astrocytes to investigate astrocyte derived extracellular vesicles.**

Katherine E. White^1^*, Hannah L. Bailey^1^, Barry S. Shaw^1^, Philippine C. Geiszler^3^, Raquel Mesquita-Ribeiro^1^, Daniel Scott^1^, Robert Layfield^1†^, Sébastien Serres^1,2^*^†^

**Supplementary information**

**Supplementary Figures**

**Supplementary Figure 1: Optimisation of serum-free culture of human primary astrocytes.** (A) Representative brightfield images of human primary astrocytes after one week (left column) or four weeks (middle column) in serum-free culture, and after 24 h treatment with 2% FBS (right column). Each row represents a serum-free media composition, as described in Supplementary Table 2. FBS treatment causes morphological changes towards a reactive phenotype as seen in serum-cultured astrocytes (black arrows). (B) Serum-cultured astrocytes after either one or five days in serum-free medium showing that once cultured in serum, morphological changes in serum-cultured astrocytes are permanent unless the cells are cryopreserved and revived in serum-free medium. Scale bars represent 100 µm for all images.

**Supplementary Figure 2: Transcription profiles of astrocyte and inflammatory markers in serum-free astrocytes are different upon acute exposure to increased concentration of FBS.** Real-time quantitative PCR analysis showing a significant increase after 2% FBS treatment for GFAP expression but not for S100β expression (*p<0.05 vs. control, and **P<0.01 vs. control, respectively, N=4-7). IL-1β expression was significantly upregulated after 2% and 10% FBS treatment (*p<0.05 vs. control, N=3-8), whilst no significant difference was identified for TNFα expression. Data represent mean ± SEM and were analysed using one-way ANOVA followed by Dunnett’s multiple comparison test.

Supplementary Figure 3: **Protein expression of GFAP and EAAT2 is different upon acute exposure to either 2% FBS or inflammatory cytokines.** Immunoblots showing a slight increase in GFAP protein expression after acute exposure to 2% FBS and a decrease in EAAT2 protein expression after acute exposure to either 2% FBS or inflammatory cytokines. These data confirmed gene expression and are represented as N=1.

Supplementary Figure 4: **Protein expression of GFAP and EAAT2 remains unchanged in serum-free astrocytes after treatment with 10 µl of serum- cultured ADEVs.**  Immunoblots showing GFAP, EAAT2 and GAPDH with no evident change in protein expression. These data confirmed gene expression and are represented as N=1.

**Supplementary Figure 5: Gene and protein expressions of HSP70 as a marker of unfolded protein response (UPR) in serum-free astrocytes upon exposure of serum-cultured ADEVs.** (A) Real-time qPCR analysis showing no change in HSP70 gene expression after treatment with either 10 or 100 µl of serum-cultured ADEVs (N=3). Data represent mean ± SEM and were analysed using one-way ANOVA followed by Dunnett’s multiple comparisons test. (B) Representative western-blots of HSP70 and GAPDH showing no change in protein expression after treatment with 10 µl of serum-cultured ADEVs (N=1).

**Supplementary Tables**

**Supplementary Table 1: Forward and reverse primer sequences used for RT-qPCR.**

| Gene of interest | Forward primer (5’-> 3’) | Reverse primer (5’-> 3’) |
| --- | --- | --- |
| B2M | AAGTGGGATCGAGACATGTAAG | GGAATTCATCCAATCCAAATGCG |
| CD49F | CTCCTGTCCCGGCTCG | CCCCACGAGCAACAGCC |
| EAAT2 | CAGGGAAAGCAACTCTAATC | CAAGGTTCTTCCTCAACA |
| GAPDH | AGCCACATCGCTCAGACAC | GCCCAATACGACCAAATCC |
| GFAP | GTGGTGAAGACCGTGGAGAT | GTCCTGCCTCACATCACATC |
| IL-10 | GACTTTAAGGGTTACCTGGGTTG | GACTTTAAGGGTTACCTGGGTTG |
| IL-1β | AGCTACGAATCTCCGACCAC | CGTTATCCCATGTGTCGAAGAA |
| NDRG2 | GAGATATGCTCTTAACCACCCG | GCTGCCCAATCCATCCAA |
| S100β | ATGTCTGAGCTGGAGAAGGC | TTCAAAGAACTGGAGAAGGC |
| TNFα | GACAAGCCTGTAGCCCATGT | TCTCAGCTCCACGCCATT |
| β-Actin | ATTGGCAATGAGCGGTTC | GGATGCCACAGGACTCCA |
| HSP70 | GTGGTGTTGAGGAAAGCAGACA | TGATCACACGTTCCACCTCATC |

**Supplementary Table 2: Composition of various media used for optimisation of serum free astrocyte culture.** Advanced or normal DMEM = Dulbecco's Modified Eagle Medium. FBS = Foetal bovine serum, AGS = astrocyte growth serum, Pen/Strep = Penicillin and Streptomycin, HB-EGF = Heparin-binding EGF-like growth factor.

| Media type | Components |
| --- | --- |
| Astrocyte medium | Astrocyte medium, 1% Pen/Strep, 1% AGS, 2% FBS |
| FBS-Free astrocyte medium (AGS AM) | Astrocyte medium, 1% Pen/Strep, 1% AGS |
| DMEM medium | DMEM with Glutamax, 1% Pen/Strep, 10% FBS |
| FBS-Free DMEM medium | DMEM with Glutamax, 1% Pen/Strep |
| Neurobasal serum-free medium (NB-27) | (50% Neurobasal media, 50% DMEM/F12), 1% Pen/Strep, 2% B27 supplement, 1% L-glutamine, 0.025% HB-EGF |
| B27 serum-free medium (DMEM B27) | DMEM/F12, 1% Pen/Strep, 2% B27 supplement, 1% L-glutamine, 0.025% HB-EGF |
| Advanced DMEM AGS medium | Advanced DMEM/F12, 1% Pen/Strep, 1% AGS, 1% L-glutamine |
| G5 serum-free medium (advanced DMEM G5) | Advanced DMEM/F12*, 1% Pen/Strep, 1% G5 supplement, 1% L-glutamine |
| B27 + G5 serum-free medium (advanced DMEM B27 G5) | Advanced DMEM/F12, 1% Pen/Strep, 1% G5 supplement, 2% B27 supplement, 1% L-glutamine |

**Supplementary Table 3: Top-ranked up-regulated RNAs in serum-cultured astrocytes compared to serum-free astrocytes.**

| **Gene** | **SF** | **SC** | **log2FC** | **pvalue** | **padj** |
| --- | --- | --- | --- | --- | --- |
| NTN4 | 466.13 | 25230.28 | 5.758 | 1.74E-298 | 1.89E-295 |
| DUSP5 | 212.69 | 5166.79 | 4.602 | 2.37E-298 | 2.48E-295 |
| PTGS1 | 522.24 | 5517.43 | 3.401 | 9.49E-298 | 9.57E-295 |
| STING1 | 98.42 | 2851.77 | 4.856 | 4.58E-284 | 4.04E-281 |
| ABLIM3 | 47.68 | 1973.15 | 5.369 | 1.37E-261 | 9.68E-259 |
| FKBP5 | 544.72 | 8974.89 | 4.043 | 1.61E-244 | 9.87E-242 |
| RGS4 | 994.19 | 38062.71 | 5.259 | 2.18E-241 | 1.28E-238 |
| SIX2 | 12.48 | 3337.13 | 8.068 | 4.41E-235 | 2.44E-232 |
| PEAR1 | 17.13 | 2487.93 | 7.180 | 5.10E-222 | 2.53E-219 |
| SRGN | 3.34 | 24667.47 | 12.861 | 1.29E-216 | 5.87E-214 |

**Supplementary Table 4: Top-ranked down-regulated RNAs in serum-cultured astrocytes compared to serum-free astrocytes.**

| **Gene** | **SF** | **SC** | **log2FC** | **pvalue** | **padj** |
| --- | --- | --- | --- | --- | --- |
| SOX2 | 11742.56 | 59.46 | -7.624 | 5.61E-306 | 7.21E-303 |
| CKB | 16180.27 | 412.25 | -5.298 | 6.32E-305 | 7.75E-302 |
| POU3F3 | 6434.95 | 90.05 | -6.157 | 6.73E-302 | 7.92E-299 |
| MLC1 | 23749.57 | 38.29 | -9.281 | 4.17E-300 | 4.71E-297 |
| A2M | 14397.63 | 26.65 | -9.088 | 3.69E-292 | 3.60E-289 |
| ARHGEF6 | 5325.28 | 355.50 | -3.906 | 2.78E-286 | 2.62E-283 |
| TNC | 65561.19 | 972.23 | -6.076 | 2.22E-285 | 2.02E-282 |
| NPAS3 | 2928.09 | 30.04 | -6.604 | 2.48E-277 | 2.12E-274 |
| FZD5 | 3676.56 | 60.57 | -5.928 | 6.17E-276 | 5.12E-273 |
| WSCD1 | 6972.01 | 70.70 | -6.417 | 1.65E-270 | 1.29E-267 |

**Supplementary Table 5: Comparison of our RNA sequencing datasets with published transcriptomics datasets.** List of astrocyte markers and of inflammatory cytokines, as reported previously (Prah et al., 2019). TNFα or LCN2 were not detected in RNA-SEQ, and IL-10 was only detected in serum-free condition at very low levels. Green arrow indicates consistent results and red arrow inconsistent results with Prah et al.


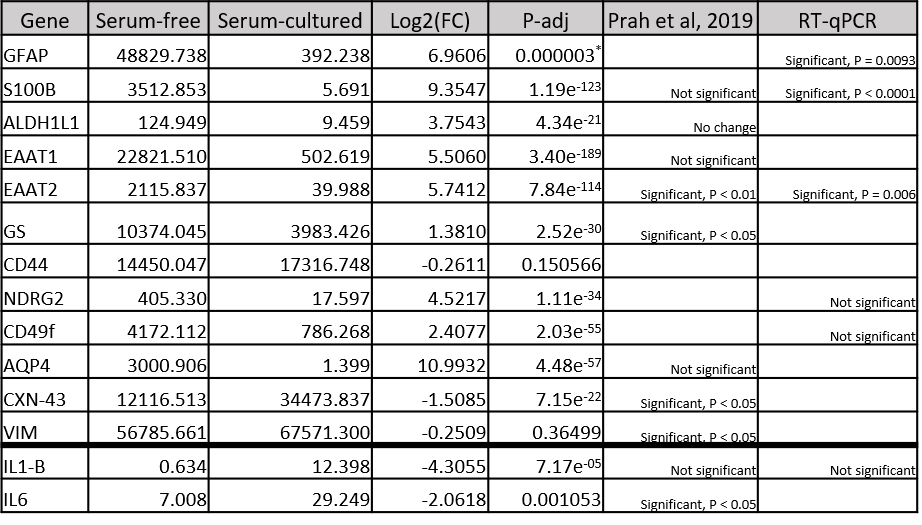


# References

Prah, J., Winters, A., Chaudhari, K., Hersh, J., Liu, R., and Yang, S.H. (2019). A novel serum free primary astrocyte culture method that mimic quiescent astrocyte phenotype. *J Neurosci Methods* 320**,** 50-63.
