# Supplementary material for: A convenient model of serum-induced reactivity of human astrocytes to investigate astrocyte-derived extracellular vesicles
Source: Front Cell Neurosci. 2024 Jun 10;18:1414142. doi: 10.3389/fncel.2024.1414142 (PMC11195030; doi:10.3389/fncel.2024.1414142)
Supplement: Supplementary file 2 [file Table_1.DOCX]

**A convenient model of serum-induced reactivity of human astrocytes to investigate astrocyte derived extracellular vesicles.**

Katherine E. White^1^*, Hannah L. Bailey^1^, Barry S. Shaw^1^, Philippine C. Geiszler^3^, Raquel Mesquita-Ribeiro^1^, Daniel Scott^1^, Robert Layfield^1†^, Sébastien Serres^1,2^*^†^

**Supplementary information**

**Key resource table**

| REAGENT OR RESOURCE | SOURCE | IDENTIFIER |
| --- | --- | --- |
| Antibodies | | |
| Rabbit anti-GFAP polyclonal antibody (Dako Omnis) | Agilent, UK | GA52461-2; RRID:AB_2811722 |
| Mouse anti-EAAT2 monoclonal antibody | Santa Cruz Biotechnology, USA | sc-365634; RRID:AB_10844832 |
| Rabbit anti-S100β polyclonal antibody (Dako Omnis) | Agilent, UK | GA50461-2;  RRID:AB_2811056 |
| Mouse anti-GAPDH monoclonal antibody | Proteintech, UK | 60004-1-Ig; RRID:AB_2107436 |
| Rabbit anti-IL-1β (D3U3E) monoclonal antibody | Cell Signalling Technology, UK | 12703; RRID:AB_2737350 |
| Rabbit anti-cleaved IL-1β (Asp116) monoclonal antibody | Cell Signalling Technology, UK | 83186; RRID:AB_2800010 |
| Rabbit anti-TNFα polyclonal antibody | Abcam, UK | ab6671; RRID:AB_305641 |
| Mouse anti-HSP70 (BRM-22) monoclonal antibody | GeneTex, USA | GTX26535;  RRID:AB_385769 |
| Mouse anti-VEGF monoclonal antibody (JH121, Invitrogen) | ThermoFisher Scientific, UK | MA5-13182; RRID:AB_10981661 |
| Goat anti-rabbit Alexa Fluor 488 antibody (Invitrogen) | ThermoFisher Scientific, UK | A-11008; RRID:AB_143165 |
| Goat anti-rabbit Alexa Fluor 568 antibody (Invitrogen) | ThermoFisher Scientific, UK | A-11011; RRID:AB_143157 |
| Goat anti-mouse Alexa Fluor 488 antibody (Invitrogen) | ThermoFisher Scientific, UK | A-11001; RRID:AB_2534069 |
| Rabbit Anti-Mouse Immunoglobulins/HRP polyclonal antibody (Dako Omnis) | Agilent, UK | P026002-2; RRID:AB_2636929 |
| Chemicals, peptides, recombinant proteins | | |
| Astrocyte medium with 2% FBS, 2% AGS supplement, and 1% penicillin/streptomycin | Caltag Medsystems, UK | Cat# SC-1801 |
| FBS-heated inactivated | Sigma-Aldrich, UK | Cat# F9665-500ML |
| Dimethyl sulfoxide | Sigma-Aldrich, UK | Cat# D8418-100ML |
| Dulbecco’s Phosphate Buffered Saline | Sigma-Aldrich, UK | Cat# D8537-500ML |
| Gibco advanced DMEM/F-12 | ThermoFisher Scientific, UK | Cat# 12634010 |
| Gibco(tm) G-5 Supplement (100X) | Fisher Scientific, UK | Cat# 10624613 |
| L-Glutamine solution | Sigma-Aldrich, UK | Cat# G7513-100ML |
| Penicillin/streptomycin | Sigma-Aldrich, UK | Cat# P0781-100ML |
| Recombinant human IL-1α | Peprotech, UK | Cat# 200-01A |
| Recombinant human TNFα | Peprotech, UK | Cat# 300-01A |
| Recombinant human C1q | Merck, UK | Cat# 204876 |
| Sodium Hydroxide, Pellets | Sigma-Aldrich, UK | Cat# 567530 |
| Glutaraldehyde solution | Sigma-Aldrich, UK | Cat# G7776 |
| Paraformaldehyde, 16% w/v aq. soln., methanol free | ThermoFisher Scientific, UK | Cat# 043368 |
| Triton X-100 | Sigma-Aldrich, UK | Cat# X100-500ML |
| Bovine Serum Albumin | Sigma-Aldrich, UK | Cat# A7906-100G |
| Vectorshield® mounting media with DAPI | 2BScientific, UK | Cat# H-2000-2ML |
| TRIzol® (Invitrogen) | ThermoFisher Scientific, UK | Cat# 15596026 |
| Superscript III reverse transcriptase (Invitrogen) | ThermoFisher Scientific, UK | Cat# 18080044 |
| PowerUp™ SYBR™ Green | ThermoFisher Scientific, UK | Cat# 15350929 |
| Sodium chloride | Sigma-Aldrich, UK | Cat# S9625 |
| Tris (Trizma ® base) | Sigma-Aldrich, UK | Cat# T6066-1KG |
| Sodium Dodecyl Sulfate (SDS) | MP Biomedicals, UK | Cat# 811032 |
| Igepal CA-630 (NP40) | Sigma-Aldrich, UK | Cat# I8896-50ML |
| EDTA disodium salt | Scientific Laboratory Supplies, UK | Cat# CHE5602 |
| Sodium deoxycholate | Sigma-Aldrich, UK | Cat# D6750 |
| Urea | Fisher Scientific, UK | Cat# 10102790 |
| Glycerol | Fisher Scientific, UK | Cat# 10795711 |
| Dithiothreitol (DTT) | Fisher Scientific, UK | Cat# BP172-5 |
| 2-mercaptoethanol | Sigma-Aldrich, UK | Cat# M6250-100ML |
| Acrylamide/Bis-acrylamide 30% sol. | Sigma-Aldrich, UK | Cat# A3699-100ML |
| AmershamTM Protran® western blotting membrane, 0.45 µm pore | Fisher Scientific, UK | Cat# 15259794 |
| Glycine | Fisher Scientific, UK | Cat# BP381-1 |
| Methanol | VWR, UK | Cat# 20847.307 |
| Tween® 20 | Fisher Scientific, UK | Cat# BP337-500 |
| Marvel milk powder | Sainsbury’s, UK | N/A |
| Laemmli sample buffer | Bio-Rad, UK | Cat# 1610747 |
| Coomassie dye | Bio-Rad, UK | Cat# 1610406 |
| Oligonucleotides | | |
| Primers used for RT-qPCR, see Table S1 | Present study | N/A |
| Critical commercial assays | | |
| Pierce BCA assay kit | ThermoFisher Scientific, UK | Cat# 23225 |
| Western Lightning Plus ECL reagents | Perkin Elmer, UK | Cat# ORT2655 |
| Novogene Advances Genomics services | Novogene, UK | N/A |
| LC-MS/MS Proteomics services | Proteomics Core Facility, Cambridge, UK | N/A |
| 4200 TapeStation system | Agilent, UK | Cat# G2991BA |
| 2000c UV/IV Spectrophotometer, nanodrop | ThermoFisher Scientific, UK | Cat# ND2000C |
| StepOne Real time PCR system | Applied Biosystems, UK | Cat# 4376599 |
| QuantStudio 5 | ThermoFisher Scientific, UK | Cat# A28574 |
| Experimental models: cells | | |
| Human Astrocytes (ScienCell) | Caltag Medsystems, UK | Cat# SC-1800 |
| Software and algorithms | | |
| SIMCA (v.18) | Sartorius Stedim Data Analytics, Sweden | Cat# UT-SS-1232 |
| FIJI software | Image J, NIH, USA | N/A |
| GraphPad prism | GraphPad Software, Inc., USA | N/A |
| DESeq2 software | N/A | (Love et al., 2014) |
| Scaffold 5 | Proteome Software, Inc, USA | N/A |
| Other | | |
| 200-mesh electron microscopy grid | EM solutions, UK | Cat# G4776 |
| 10 kDa MWCO protein concentrator | ThermoFisher Scientific, UK | Cat# 88528 |
| qEV original 70 nm column | Izon, France | Cat# ICO-70 |
| 3 kDa MWCO protein concentrator | Merck, UK | Cat# UFC5003 |
