# Supplementary figures and images for: A convenient model of serum-induced reactivity of human astrocytes to investigate astrocyte-derived extracellular vesicles
Source: Front Cell Neurosci. 2024 Jun 10;18:1414142. doi: 10.3389/fncel.2024.1414142 (PMC11195030; doi:10.3389/fncel.2024.1414142)

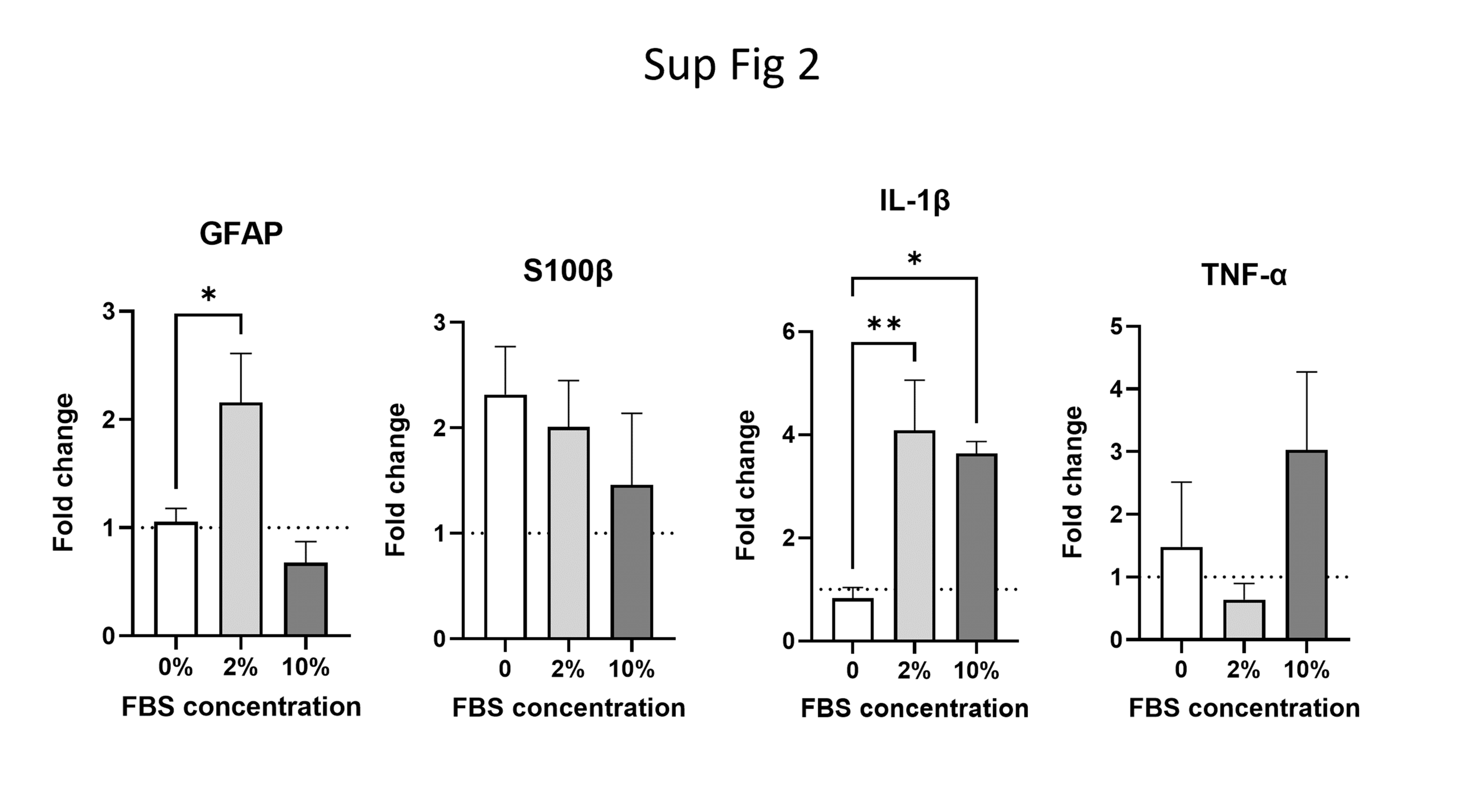

Supplement: Supplementary file 5 [file Image_2.TIF]

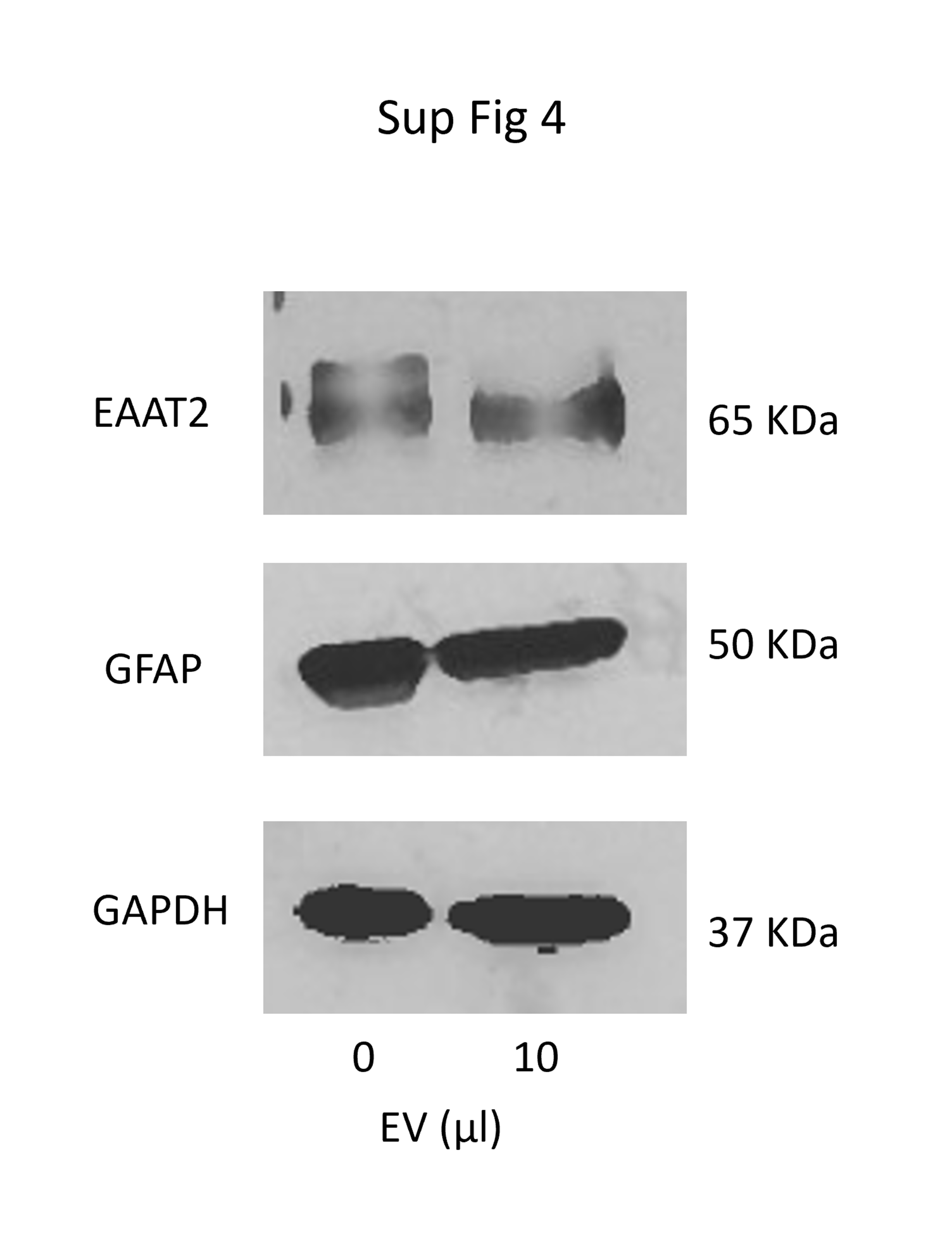

Supplement: Supplementary file 7 [file Image_4.TIF]

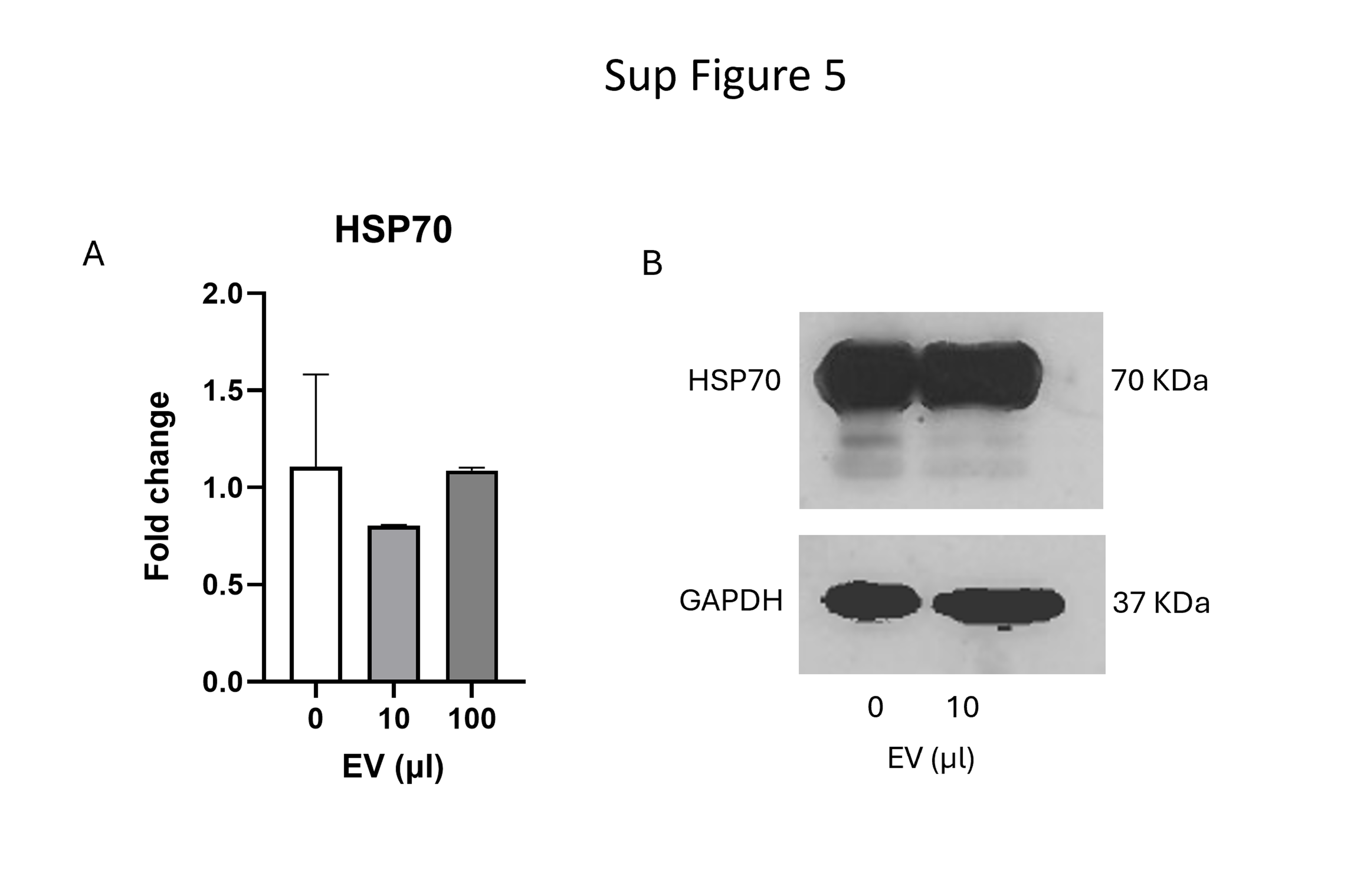

Supplement: Supplementary file 8 [file Image_5.TIF]
